# Heterogeneous Tumor Subpopulations Cooperate to Drive Invasion

**DOI:** 10.1016/j.celrep.2014.06.045

**Published:** 2014-07-24

**Authors:** Anna Chapman, Laura Fernandez del Ama, Jennifer Ferguson, Jivko Kamarashev, Claudia Wellbrock, Adam Hurlstone

**Affiliations:** 1 Faculty of Life Sciences, The University of Manchester, Oxford Road, Manchester, M13 9PT, UK; 2 Department of Dermatology, University Hospital Zürich, Gloriastrasse 31, 8091 Zürich, Switzerland

## Abstract

Clonal selection and transcriptional reprogramming (e.g., epithelial-mesenchymal transition or phenotype switching) are the predominant theories thought to underlie tumor progression. However, a “division of labor” leading to cooperation among tumor-cell subpopulations could be an additional catalyst of progression. Using a zebrafish-melanoma xenograft model, we found that in a heterogeneous setting, inherently invasive cells, which possess protease activity and deposit extracellular matrix (ECM), co-invade with subpopulations of poorly invasive cells, a phenomenon we term “cooperative invasion”. Whereas the poorly invasive cells benefit from heterogeneity, the invasive cells switch from protease-independent to an MT1-MMP-dependent mode of invasion. We did not observe changes in expression of the melanoma phenotype determinant MITF during cooperative invasion, thus ruling out the necessity for phenotype switching for invasion. Altogether, our data suggest that cooperation can drive melanoma progression without the need for clonal selection or phenotype switching and can account for the preservation of heterogeneity seen throughout tumor progression.

## Highlights

• Inherently invasive and poorly invasive melanoma subpopulations co-invade
• Leader cells are inherently invasive and provide MT1-MMP and deposit ECM
• Follower cells affect the mode of invasion and thereby elicit protease dependency
• Cooperative invasion bypasses phenotype switching and maintains tumor heterogeneity

## Graphical Abstract

**Figure:**
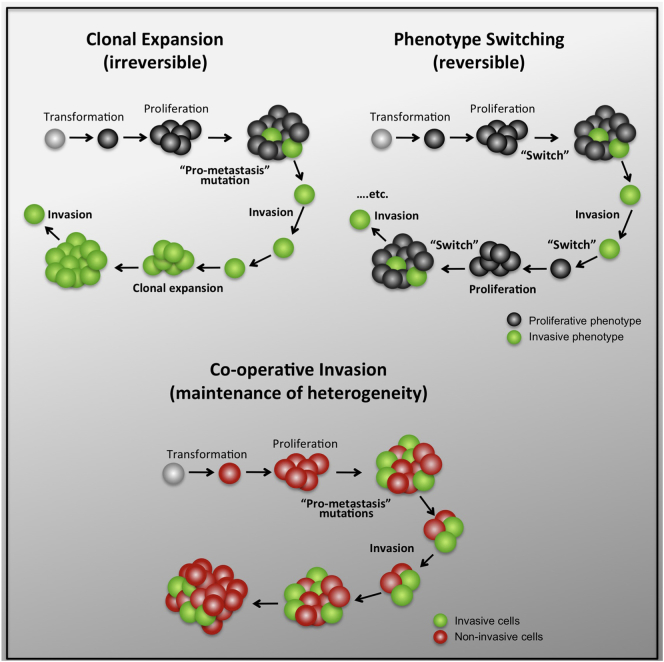

## Teaser

Tumor heterogeneity has been viewed as the outcome of adaptive competition or differential transcriptional reprogramming (phenotype switching). Alternatively, heterogeneity may reflect a division of labor between tumor cell subpopulations with complementary acquired characteristics. Chapman et al. now show reciprocal interactions between heterogeneous melanoma cell subpopulations that lead to cooperative invasion without competition and without phenotype switching. Thus, cooperative behavior emerges as a property of heterogeneity relevant for tumor biology and worth targeting therapeutically.

## Introduction

Tumors comprise subpopulations of transformed cells that are genotypically or phenotypically divergent. In melanoma, cell subpopulations have been characterized that differ in gene expression profiles, proliferation rates, and invasiveness; leading to the definition of so-called proliferative and invasive phenotypes that correlate with relatively high and low expression, respectively, of the melanocyte lineage determinant MITF (Hoek et al., 2006, 2008). However, whether interactions between heterogeneous melanoma cell subpopulations contribute to invasion and metastasis is unknown. To study the potential significance of heterogeneity for the dissemination of melanoma cells, we developed a xenograft model in zebrafish embryos that allows monitoring of local invasion with high resolution. To represent genotypes relevant to melanoma we selected melanoma cell line pairs that harbor the V600E BRAF mutation, the most common mutation present in melanoma (Wellbrock and Hurlstone, 2010), but differ in their expression of MITF, and as a consequence the individual cell lines are either inherently invasive (MITF^low^) or poorly invasive (MITF^high^). We find that these divergent cell lines communicate reciprocally and cooperate to invade collectively dependent on protease activity and fibronectin deposition and without altering MITF expression.

## Results

### Heterogeneity Confers Invasive Properties on Individually Poorly Invasive Melanoma Cells

**Figure 1:**
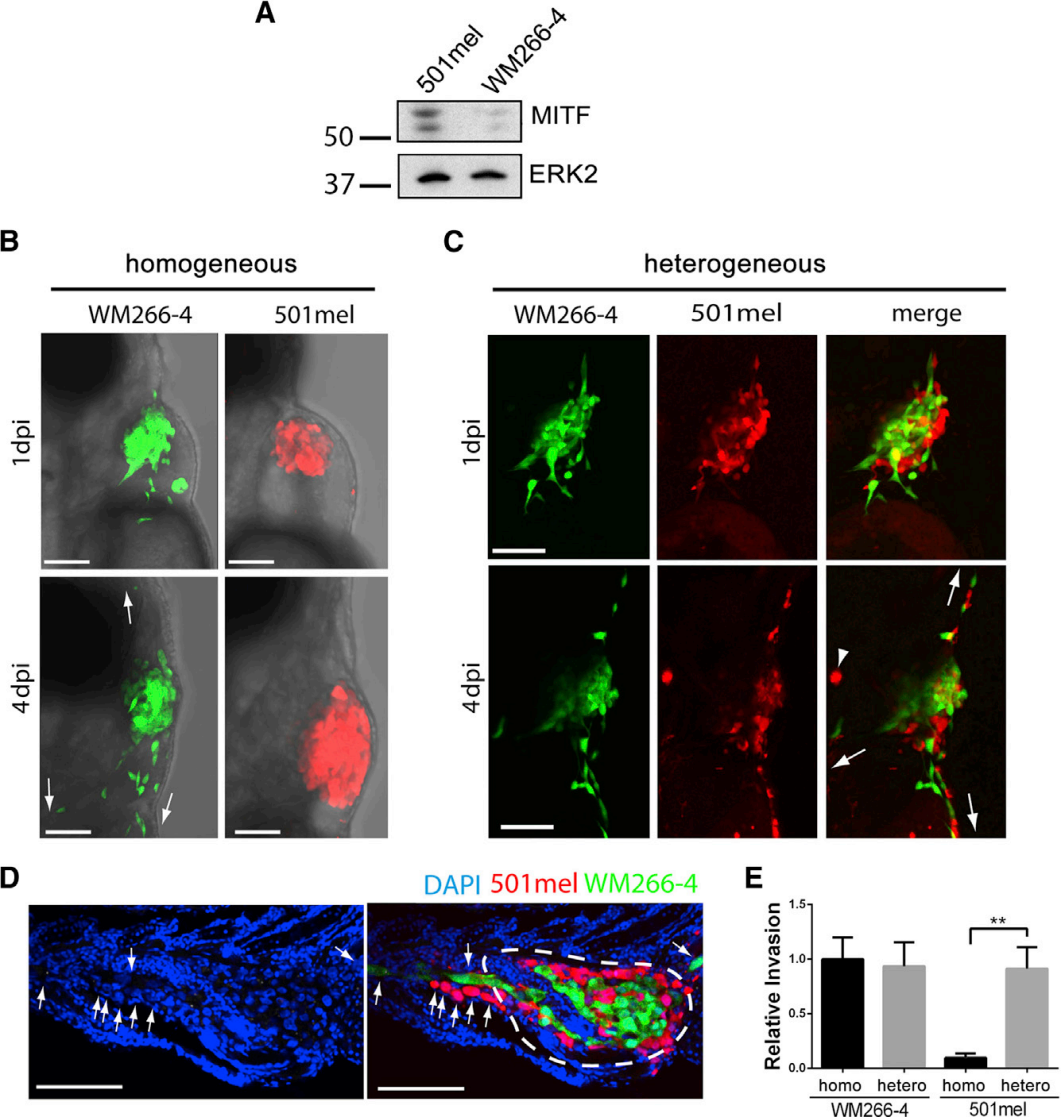
Heterogeneity Results in Cooperative Invasion (A) Western blot showing MITF expression in WM266-4 and 501mel cells. (B) Homogeneous xenografts imaged at 1 (upper) and 4 days (lower) postinjection (dpi). (C) Heterogeneous xenografts imaged at 1 (upper) and 4 dpi (lower). Arrows indicate directions of invasion; arrowhead indicates autofluorescence. (D) Section from engrafted embryo indicating primary tumor site (white dashed line) and infiltrating melanoma cells (white arrows). Scale bars represent 100 μm. (E) Quantitation of invasion depicted in (A) and (B); mean ± SEM; Kruskal-Wallis test followed by Dunn’s multiple comparisons test; ^∗∗^p < 0.01; n ≥ 26 from three independent experiments. See also Figure S1.

Invasive MITF^low^ WM266-4 cells or poorly invasive MITF^high^ 501mel cells (Figure 1A; Arozarena et al., 2011; Ohanna et al., 2011; Rozenberg et al., 2010) were injected into the pericardial cavity of zebrafish embryos at 48 hr postfertilization (Figures S1A and S1B available online). Both melanoma cell lines (WM266-4-GFP and 501mel-mCherry) aggregated rapidly and anchored to the body wall to form tumor-like masses (Figures 1B and S1C). As anticipated, WM266-4 cells invaded efficiently, but 501mel cells displayed little invasion (Figure 1B). Strikingly, however, in a heterogeneous situation, when WM266-4 and 501mel cells were present in equal ratios within the xenograft, the invasion of 501mel cells increased markedly (Figure 1C). In tissue sections of engrafted zebrafish embryos, infiltrating tumor cells were found migrating away from the primary site through solid tissue (Figure 1D). We enumerated the invading cells located outside the pericardium (dashed line in Figure 1D). This confirmed that the invasion of 501mel cells increased to levels similar to WM266-4 cells (Figure 1E). This striking behavior was also observed for another pair of MITF^low^ and MITF^high^ melanoma cell lines—UACC62 and 888mel cells, respectively—(Figures S1D–S1F), suggesting this may be a general phenomenon. Thus, melanoma cells that display divergent invasive phenotypes in isolation interact symbiotically under heterogeneous circumstances, a phenomenon that we describe as “cooperative invasion”.

### Cooperative Invasion Requires the Activity of MT1-MMP

Further analysis revealed that WM266-4 cells were significantly more likely to be the leading cell of an invasive file (Figure S2A). Live imaging confirmed that over time WM266-4 cells lead files of invading cells (Figure S2B). Such behavior has been described for tumor-associated fibroblasts, which contribute to the matrix metalloproteinase (MMP)-mediated degradation of the extracellular membrane (ECM), thereby enabling epithelial cancer cells to invade (Gaggioli et al., 2007). Real-time PCR revealed significantly higher expression of the three most prominent cancer related MMPs—MMP1, MMP2 and MT1-MMP—in WM266-4 cells compared to 501mel cells (Figure S2C), suggesting that during cooperative invasion, WM266-4 cells could adopt a role similar to fibroblasts.

**Figure 2:**
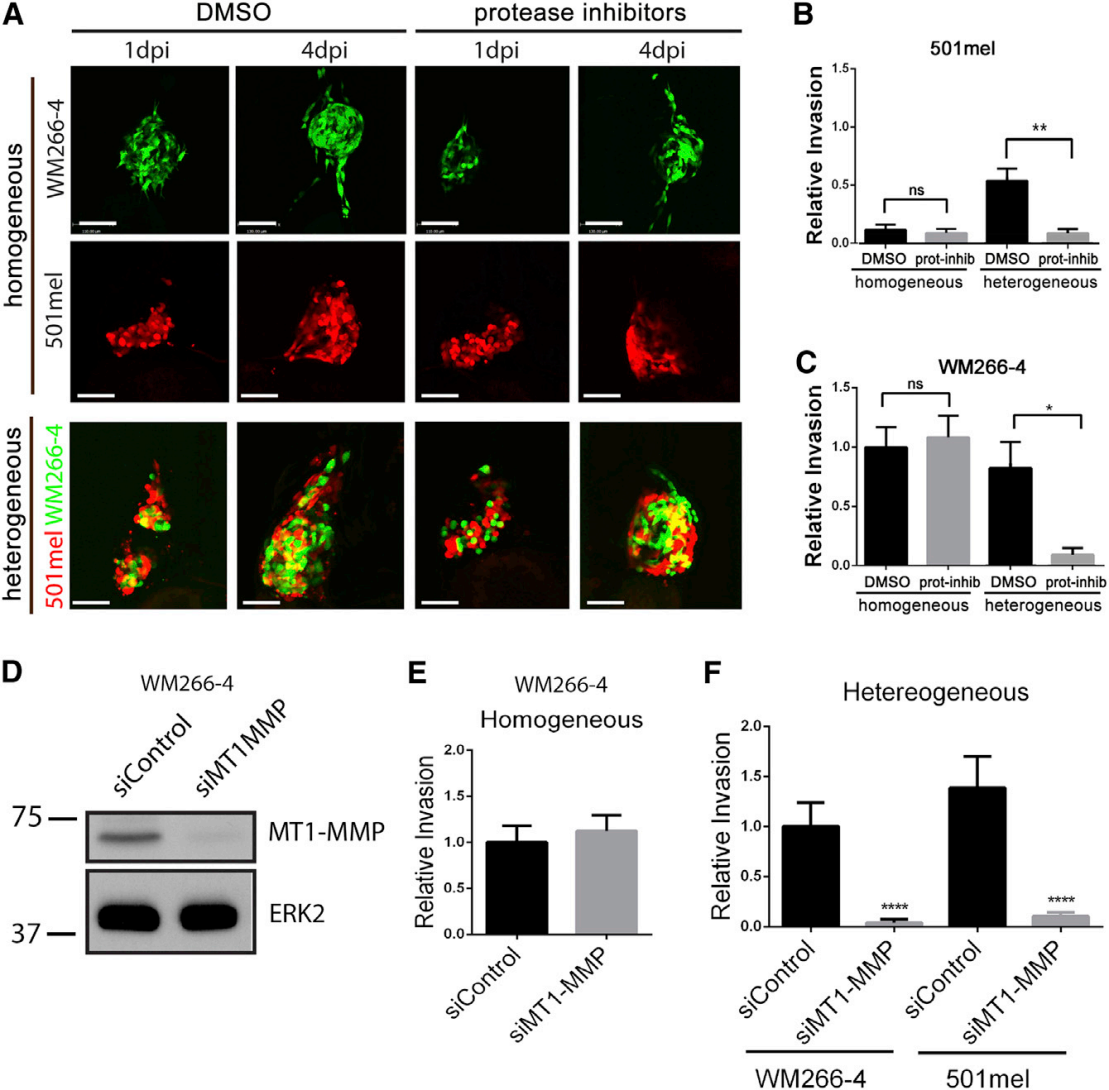
MMP Inhibition Suppresses Cooperative Invasion (A) Homogeneous (upper) or heterogeneous (bottom) xenografts were treated with either the vehicle control DMSO (left) or protease inhibitor cocktail (right). Scale bars represent 100 μm. (B) Quantitation of 501mel invasion depicted in (A); mean ± SEM; Kruskal-Wallis test followed by Dunn’s multiple comparisons test; ^∗∗^p < 0.01; n ≥ 9 from three independent experiments. (C) Quantitation of WM266-4 invasion depicted in (A); mean ± SEM; Kruskal-Wallis test followed by Dunn’s multiple comparisons test; ^∗^p < 0.05; n ≥ 13 from three independent experiments. (D) Western blot showing MT1-MMP expression in WM266-4 transfected with either control or MT1-MMP specific siRNA. (E) Quantitation of invasion of WM266-4 cells in homogeneous xenografts wherein WM266-4 cells have been transfected with either control or MT1-MMP-specific siRNA; mean ± SEM; Mann-Whitney test; n ≥ 21 from three independent experiments. (F) Quantitation of invasion of WM266-4 and 501mel in heterogeneous xenografts wherein WM266-4 cells have been transfected with either control or MT1-MMP specific siRNA; mean ± SEM; Kruskal-Wallis test followed by Dunn’s multiple comparisons test; ^∗∗∗∗^p < 0.0001; n ≥ 24 from three independent experiments. See also Figure S2.

We assessed the relevance of protease activity for cooperative invasion by incubating engrafted embryos with a previously described cocktail of protease inhibitors (Sahai and Marshall, 2003; Wolf et al., 2003). This had no effect on homogeneous 501mel xenografts, where no invasion occurred in any case (Figures 2A and 2B). However, the invasion of 501mel cells in heterogeneous xenografts was almost completely blocked in the presence of the inhibitor mix (Figures 2A and 2B). This indicates that the proteolytic cleavage of ECM is necessary for the acquired invasion of 501mel cells. Homogeneous WM266-4 xenografts showed no difference in relative invasion when treated with the protease inhibitors compared to DMSO (Figures 2A and 2C), suggesting that WM266-4 cells can use a proteolytic-independent mechanism of invasion, as has been described elsewhere (Sahai and Marshall, 2003; Wolf et al., 2003). Surprisingly, however, in heterogeneous xenografts treated with the protease inhibitor mix WM266-4 cells showed a dramatic decrease in invasion (Figures 2A and 2C). Suppression of cooperative invasion but not invasion of homogeneous WM266-4 cells was also observed when the pan-MMP inhibitor GM6001 was used alone, albeit at higher concentration (Figures S2D and S2E). Because MT1-MMP is a major regulator of protease-driven invasion (Sabeh et al., 2004), we depleted MT1-MMP expression in WM266-4 cells using RNAi; this efficiently suppressed cooperative invasion, although again did not affect the invasion of homogeneous WM266-4 cells (Figures 2D–2F and S2F). Further corroborating the importance of MT1-MMP for cooperative invasion, invasive UACC62 cells also express significantly more MT1-MMP than poorly invasive 888mel cells (Figure S1F). Thus, we not only identify a crucial role for MT1-MMP in cooperative invasion, but also demonstrate that it is tumor cell protease activity rather than host protease activity that is required for cooperative invasion.

**Figure 3:**
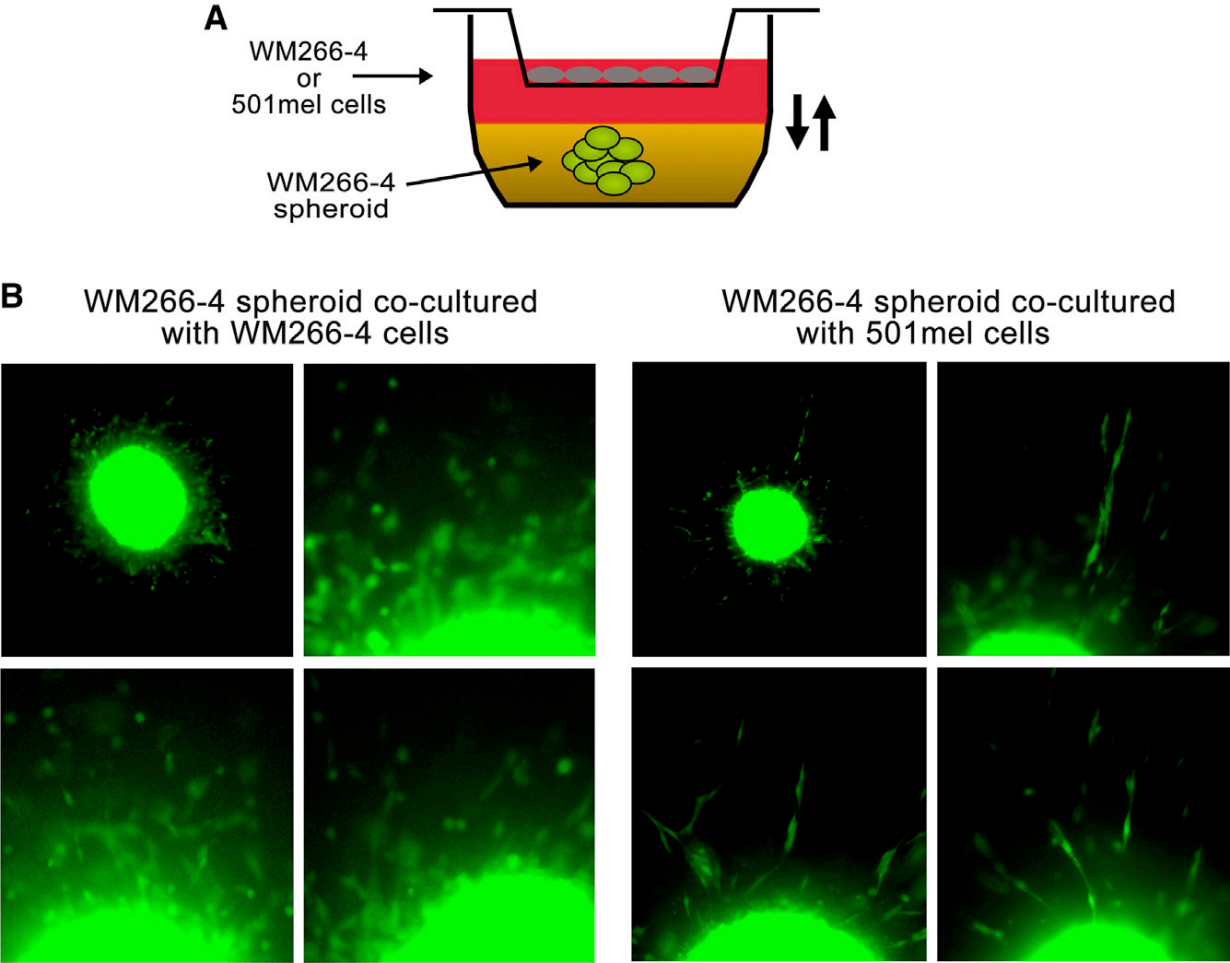
A Diffusible Factor Emanating from 501mel Cells Modulates WM266-4 Cell Response to Protease Inhibitors (A) Cartoon depicting experimental set-up, with WM266-4 spheroids being cocultured either with autologous cells or heterologous cells in porous transwells. (B) Representative images of WM266-4 spheroids cocultured either with WM266-4 or 501mel cells in the presence of a cocktail of protease inhibitors.

The preceding experiments indicated that the presence of 501mel cells suppressed the protease-independent invasive potential of WM266-4 cells. To further investigate the mechanism of crosstalk, we cultured WM266-4 cells as spheroids embedded in pepsin-extracted bovine collagen in the absence or presence of 501mel cells and added protease inhibitors to the culture system (Figure 3A). We found that under these conditions, homogeneous WM266-4 cells invaded the matrix singly with a predominantly rounded morphology (Figure 3B), in line with previously published data (Arozarena et al., 2011; Sahai and Marshall, 2003). However, exposure to soluble factors derived from 501mel cells resulted in a reduction of invasion and a switch to an elongated mode of invasion (Figure 3B), which is known to be protease dependent.

### Cooperative Invasion Involves Changes in the ECM

**Figure 4:**
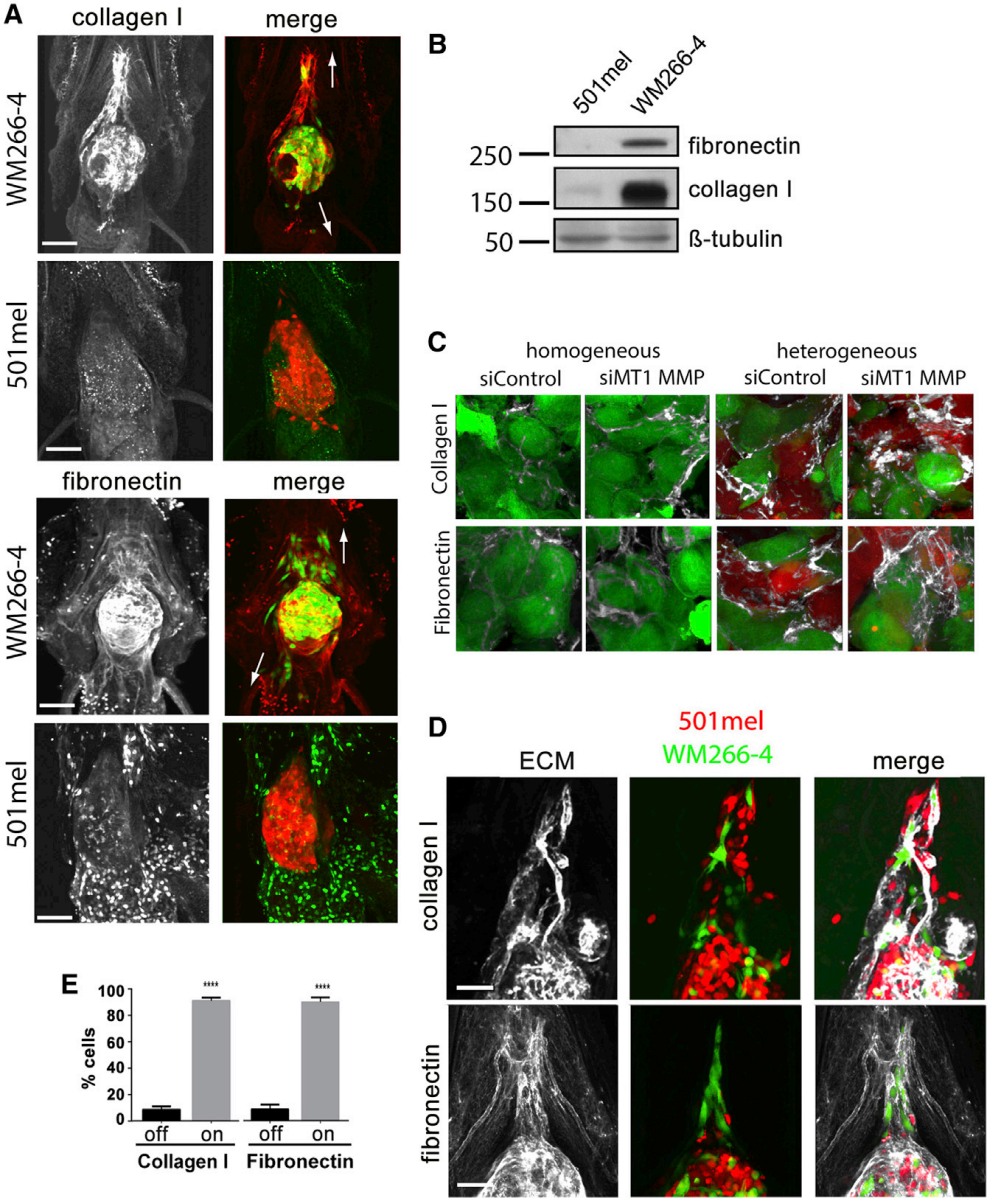
ECM Proteins Correlate with Invasiveness (A) Expression of ECM components collagen I and fibronectin in engrafted zebrafish 4 dpi; arrows indicate direction of invasion. (B) Western blot showing collagen I and fibronectin expression in WM266-4 and 501mel cells. (C) Collagen I (upper) and fibronectin (lower) in homogeneous compared to heterogeneous xenografts that are further treated with either DMSO or GM6001. (D) Invasive WM266-4 and 501mel follow collagen I (upper) and fibronectin (lower) tracks radiating out from the tumor. (E) Quantitation of ECM association. Cells were scored as being in touch “on” with collagen I or fibronectin strands or not “off.” Mean ± SEM; unpaired Student’s t test (collagen) and Mann-Whitney test (fibronectin); ^∗∗∗∗^p < 0.0001; n ≥ 18 from three independent experiments. Scale bars represent 100 μm. See also Figure S3.

MMPs and fibronectin are coexpressed in melanoma cells (Kamoshida et al., 2013), supporting the idea that ECM deposition and degradation are closely coordinated. Also, fibronectin is associated with melanoma progression (Gaggioli et al., 2005) and can regulate the organization of type I collagen fibrils (Sottile and Hocking, 2002). To analyze the involvement of type I collagen and fibronectin in cooperative invasion, we first performed whole-mount immunofluorescence staining on homogeneous WM266-4 and 501mel xenografts: WM266-4 xenografts were surrounded by abundant type I collagen and fibronectin, but this was not detectable in 501mel xenografts (Figure 4A). Western blotting revealed a strong expression of both ECM proteins in WM266-4 cells, but barely detectable expression in 501mel cells (Figure 4B), suggesting that the observed ECM in the xenografts is derived from WM266-4 cells.

Intriguingly, ECM detected in heterogeneous xenografts was more abundant than in WM266-4 homogeneous xenografts (Figures 4C and S3A–S3C), a further indication of reciprocal communication between the two subpopulations. Additionally, coculturing WM266-4 cells with 501mel cells in a transwell system resulted in increased ECM protein expression in both cell lines (Figure S3D), suggesting the involvement of diffusible factors in this communication. Moreover, ECM deposition was further augmented in xenografts comprising WM2664-cells depleted for MT1-MMP or treated with protease inhibitors, and matrix components were more diffuse (Figures 4C and S3A–S3C). However, protease inhibition did not lead to further collagen I and fibronectin induction in our in vitro coculture system (Figure S3D). This implies that the increased amount and disorder observed because of protease inhibition in vivo (Figures 4C and S3A–S3C) may be due to modulating ECM turnover rather than expression.

### Cooperative Invasion Depends on Fibronectin

In addition to the ECM around the WM266-4 tumor mass, we also observed ECM around invading cells (Figure 4A). Close examination revealed fibers of fibronectin and collagen I radiating away from the xenograft (Figure S3E), typical of the arrays of collagen fibers detected around breast tumors that are associated with files of invading cells (Provenzano et al., 2006). In heterogeneous xenografts, we observed that both WM266-4 and 501mel cells appear to migrate in close connection to the tracks of ECM (Figure 4C). Quantitation showed that during cooperative invasion, cells were found predominantly on these collagen and fibronectin tracks (Figure 4D).

**Figure 5:**
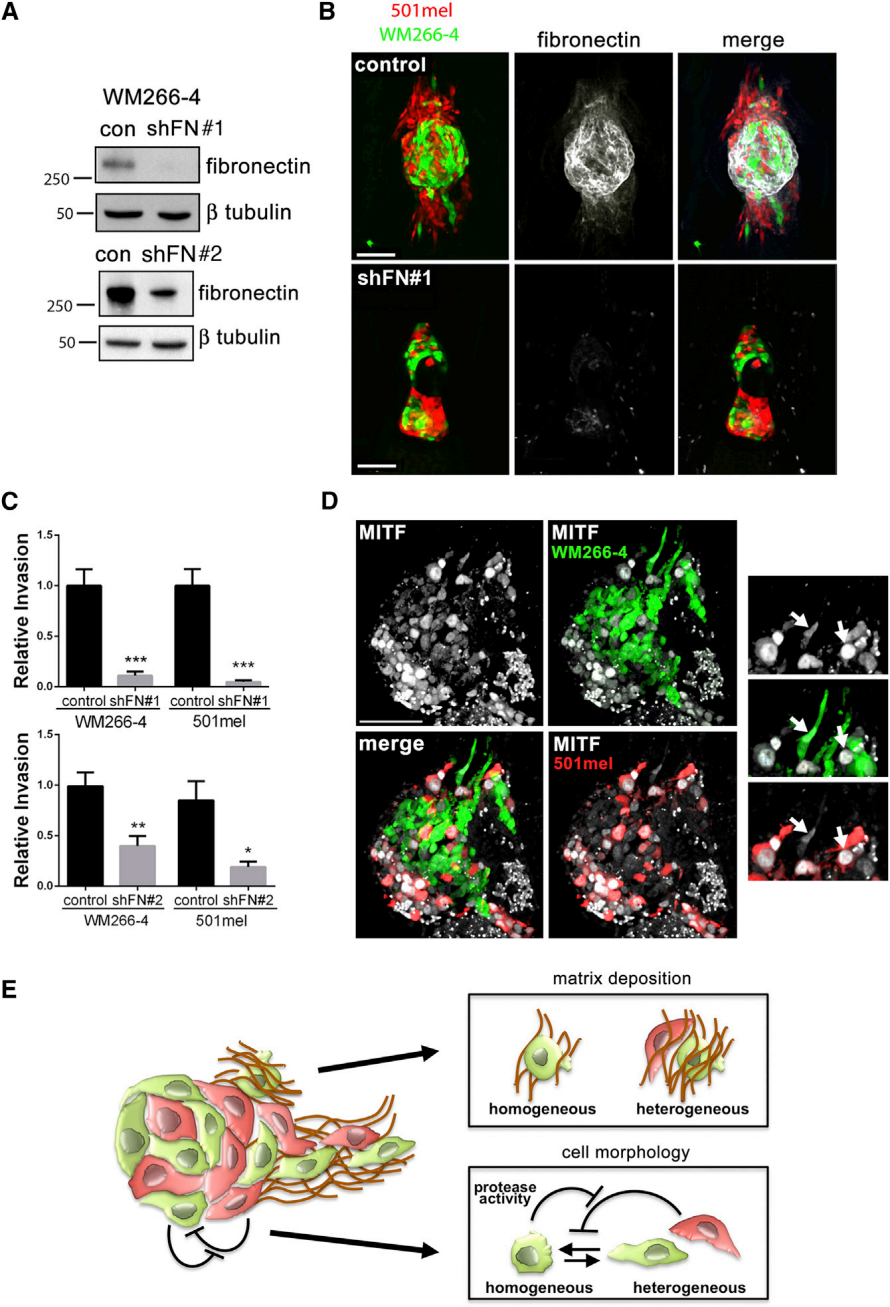
Fibronectin Is Essential for Cooperative Invasion; Invasive Primary Melanoma Cells Are Also Heterogeneous (A) Western blot showing stable knockdown of fibronectin in WM266-4 GFP shFN cells. #1 and #2 are clones expressing independent shRNA targeting fibronectin. Control (con) cells express an irrelevant shRNA. (B) Fibronectin associated with heterogeneous xenografts comprising either control WM266-4 cells (upper) or WM266-4 shFN#1 cells (lower). (C) Quantitation of invasion of 501mel and WM266-4 cells from heterogeneous xenografts comprising either control WM266-4 cells, WM266-4 shFN#1, or WM266-4 shFN#2 cells. Mean ± SEM; Mann-Whitney test; ^∗^p < 0.05, ^∗∗^p < 0.001, ^∗∗∗^p < 0.001; n ≥ 18 from three independent experiments. (D) MITF immunofluorescence in frozen sections of heterogeneous xenografts. Arrows indicate high and low MITF fluorescence intensity in invading cells. (E) Model depicting the reciprocal interactions underlying cooperative invasion. See also Figures S4 and S5.

To test whether ECM deposition was essential for cooperative invasion, we generated WM266-4 cells expressing low levels of fibronectin through RNA interference (WM266-4 shFN#1 and shFN#2 cells). The reduction of fibronectin production was confirmed in vitro with western blotting (Figure 5A). In vivo, the presence of WM266-4 shFN cells led to a significant loss of fibronectin deposition around either homogeneous or heterogeneous xenografts when compared to xenografts containing control WM266-4 cells (Figures 5B and S4A), confirming that the fibronectin associated with the xenograft was derived largely from WM266-4 cells. When we analyzed collagen deposition, we found that fibronectin knockdown did not affect the expression of collagen I in WM266-4 or its deposition in vivo (Figures S4B and S4C), suggesting that collagen deposition was independent of fibronectin. When WM266-4 shFN cells were injected as a heterogeneous mixture with 501mel cells, the invasion of 501mel cells was dramatically reduced when compared to heterogeneous xenografts containing WM266-4 control cells (Figures 5B and 5C), and more so for shFN#1 than shFN#2 cells reflecting the magnitude of fibronectin knockdown. This suggested that cooperative invasion of 501mel cells requires the presence of WM266-4 generated fibronectin tracks. In line with such a role for fibronectin, invasive UACC62 cells also express significantly more fibronectin than 888mel cells (Figure S1F). Interestingly, the reduction in fibronectin expression appeared to not affect the invasion of WM266-4 cells in homogeneous xenografts (Figure S4D). However, their invasion was impaired in heterogeneous tumors when fibronectin deposition was suppressed (Figures 5B and 5C), further supporting a reciprocal communication between the individual melanoma cell subpopulations in a heterogeneous setting.

### MITF^high^ and MITF^low^ Cells Are Present in the Invasive Front of Tumors

One of the major determinants of melanoma heterogeneity is the regulation of MITF expression by the tumor microenvironment (Hoek et al., 2008). Further, a microenvironment-induced switch to a MITF^low^ phenotype is thought to drive tumor invasion (Carreira et al., 2006). Although MITF expression correlates with the invasiveness of individual cell populations (see Figure 1B and Figure S1D), the cooperative invasion observed in heterogeneous xenografts (see Figures 1C and S1D) suggested that both MITF^high^ and MITF^low^ cells could contribute to tumor invasion. Indeed, immunofluorescence to detect MITF expression in heterogeneous xenografts revealed that MITF^high^ and MITF^low^ cells invade together (Figure 5D), indicating that MITF downregulation is not required for cooperative invasion. To extend these findings into the clinical setting, human melanoma biopsies were also examined for MITF expression. Consistent with MITF heterogeneity being present in invading cells, MITF staining revealed that MITF^high^ and MITF^low^ cells coexist in groups of melanoma cells invading the dermis (Figure S5A), which is also apparent in other published data (King et al., 1999) and in biopsy samples displayed in the Human Protein Atlas http://www.proteinatlas.org (Uhlen et al., 2010; Figure S5B).

## Discussion

Tumors usually display a high degree of genotypic and phenotypic heterogeneity, but the impact of heterogeneity on tumor progression is not understood. Using a novel xenograft model, we explored the possibility of phenotypically divergent melanoma cells cooperating during the first steps of tumor progression by analyzing their invasive behavior. We describe here what we call cooperative invasion, during which heterogeneous melanoma cell subpopulations interact reciprocally and mobilize collectively.

Our data suggest that both proteolytic activity as well as ECM deposition are necessary for cooperative invasion because disruption of either completely abrogates invasion in heterogeneous xenografts. Moreover, we show that protease activity is required to organize rather than simply degrade the ECM. It is known that the ECM within a tumor is distinct from normal tissue, due not only to alterations in composition, but also through increased ECM stiffness (Levental et al., 2009). Alterations in tension alone can be sufficient to alter tumor cell invasion via integrin activation (Friedland et al., 2009). Thus, WM266-4-produced fibronectin could trigger changes in integrin signaling in 501mel cells, or provide tracks serving as paths for invading 501mel cells. However, the full mechanism of this role of fibronectin is yet to be resolved.

Another important finding from our study is that the mode of invasion of WM266-4 cells switches from protease independent in homogeneous xenografts to MT1-MMP dependent in heterogeneous xenografts and resembles what has been described as collective invasion of chains of invading cells (Wolf et al., 2007). We hypothesize that 501mel cells secrete factors that induce an elongated morphology in WM266-4 cells thereby constraining them to adopt a protease-driven leader role in invasion; however, this soluble “switch factor” can be cleaved by proteases secreted by WM266-4 cells, allowing them to use “rounded” invasion when the “switch factor” is sufficiently neutralized. Potentially the same or possibly alternative secreted factors emanating from 501mel cells also augment ECM density in heterogeneous xenografts, which would too promote a protease-dependent invasion mode (Wolf et al., 2013). Together, our data highlight that when cells cooperate, the underlying cell-cell communications produce reciprocal effects on the individual subpopulations (see Figure 5E for a model). Identifying the underlying mechanisms will be crucial if we are to fully understand the impact of heterogeneity on invasion. Moreover, the reciprocal communication underlying cooperation may itself be a therapeutic target.

Tumor progression is a complex cascade of events requiring tumor cells to detach from the primary tumor, invade locally, intravasate, survive in circulation, extravasate, and finally colonize distant organs. How cancer cells acquire all these capabilities has been the subject of considerable speculation. Historically, competition between genetically divergent cancer cell variants leading to expansion of the “fittest” clone was thought to drive metastasis (Greaves and Maley, 2012). However, in multiple tumor types, secondary tumors retain the heterogeneity of primary tumors, display remarkably similar gene expression, and have very similar constellations of mutations (Ramaswamy et al., 2003; Vakiani et al., 2012; Vignot et al., 2013; Walter et al., 2012), challenging the clonal expansion model.

An alternative theory proposes that cancer cells switch behavior reversibly in response to transient changes in gene expression, which are triggered by microenvironmental cues (e.g., hypoxia or inflammation). As such, in carcinomas, reversible transitions between epithelial and mesenchymal phenotypes (EMT ↔ MET) throughout tumor progression might explain some of the similarity seen in primary and secondary tumors (Scheel and Weinberg, 2012). In melanoma, phenotype switching is thought to follow altered MITF expression (Hoek and Goding, 2010). In both examples, a switch is proposed to occur to generate different phenotypes with only one phenotype being compatible with a particular stage of tumor progression. However, this is contradicted by our observation that the invasive front in both our xenograft model and patient biopsies comprise heterogeneous cells. Moreover, melanoma cell clusters circulating in patient blood were shown to express MITF heterogeneously (Khoja et al., 2014).

We believe that a process that maintains tumor heterogeneity throughout progression based on cancer cell cooperation better explains the preservation of heterogeneity in metastases. In support of this notion, other examples have emerged that demonstrate cooperative behavior among cancer cells. For instance, in *Drosophila*, separate clones of cells bearing RasV12 and *scribble* mutations can interact to propagate Jnk signaling resulting in neoplasia (Wu et al., 2010). In a mouse breast cancer model driven by MMTV-Wnt1, tumors were identified containing distinct basal *Hras*^*mut*^* Wnt1*^low^ and luminal *Hras*^*wt*^* Wnt1*^high^ subclones, both of which were required for efficient tumor propagation (Cleary et al., 2014). Furthermore, carcinoma cells that had undergone EMT can cooperate with cancer cells that still possess all epithelial traits to induce lung metastasis (Tsuji et al., 2008), and EMT and non-EMT cells were also discovered coexisting in circulating tumor cell clusters (Hou et al., 2012; Yu et al., 2013). Symbiotic interactions between individual cancer cell subpopulations may well affect drug responses (as we have seen with protease inhibitors), and, in the future, tumor heterogeneity should be addressed in preclinical stages of drug development.

## Experimental Procedures

### Cell Culture

Cells were maintained at 37°C/5% CO_2_ in Dulbecco's modified Eagle's medium (Sigma) supplemented with 10% fetal bovine serum (Sigma) and 0.5% penicillin-streptomycin (Sigma). Prior to injection, UACC62 cells were stained with CellTrace CFSE Cell Proliferation Kit (Invitrogen). WM266-4 shFN cells were generated with the Block-iT Pol II miR RNAi expression vector kit (Invitrogen). MT1-MMP knockdown in WM266-4 cells was achieved by transfecting 2nM siRNA against MT1-MMP (Dharmacom) using INTERFERin (Polyplus Transfection) transfection reagent.

### Xenograft Assay

All animal studies described within were approved by The University of Manchester Ethical Review Board and performed according to UK Home Office regulations. Suspended cells were injected into the pericardial cavity of 48 hr postfertilization zebrafish embryos. Engrafted embryos were maintained at 34°C for 4 days. As needed, embryos were treated at 1 day postinjection (dpi) with DMSO (Sigma) or a cocktail of protease inhibitors or GM6001 alone (for 72 hr). At 4 dpi, embryos were fixed in 4% paraformaldehyde (PFA, Sigma) in PBS at 4°C overnight.

### Whole-Mount Immunofluorescence

Embryos were incubated with anti-collagen I (1:800 dilution, rabbit polyclonal, 1:4,000 dilution; Rockland Immunochemicals) and anti-fibronectin (1:1,000 dilution, mouse monoclonal Ab6328; Abcam) antibodies and then with secondary Alexa Fluor antibodies: anti-mouse 594, anti-mouse 633, anti-rabbit 594, and anti-rabbit 633 (1:150 dilution, Invitrogen).

### Microscopy and Analysis

Tumors were imaged at 1 and 4 dpi using a Leica TCS SP5 AOBS upright confocal (Leica Microsystems). Z stacks were processed using Volocity software (Perkin Elmer). All experiments were performed a minimum of three times. Relative invasion is defined as the average number of cells located outside the pericardial cavity at 4 dpi normalized to the average number for the control group. All statistical analysis was performed using GraphPad Prism version 5 (GraphPad Software).

## Author Contributions

A.C. helped design and performed the majority of experiments (with support from L.F.D.A. and J.F.) and analyzed data; J.K. performed immunostaining for MITF on patient samples and acquired images; and C.W. and A.H. conceived the study, designed experiments, and analyzed data. All authors contributed to interpreting results and writing the paper.

## Supplemental Information

Document S1. Supplemental Experimental Procedures and Figures S1–S5

Document S2. Article plus Supplemental Information
