# Supplementary material for: Heterogeneous Tumor Subpopulations Cooperate to Drive Invasion
Source: Cell Rep. 2014 Jul 24;8(3):688–95. doi: 10.1016/j.celrep.2014.06.045 (PMC4542310; doi:10.1016/j.celrep.2014.06.045)
Supplement: Document S1. Supplemental Experimental Procedures and Figures S1–S5 [file mmc1.pdf]

SUPPLEMENTAL INFORMATION

Heterogeneous tumour-subpopulations co-operate to drive invasion

Anna Chapman, Laura Fernandez del Ama, Jennifer Ferguson, Jivko Kamarashev, Claudia Wellbrock, and Adam Hurlstone

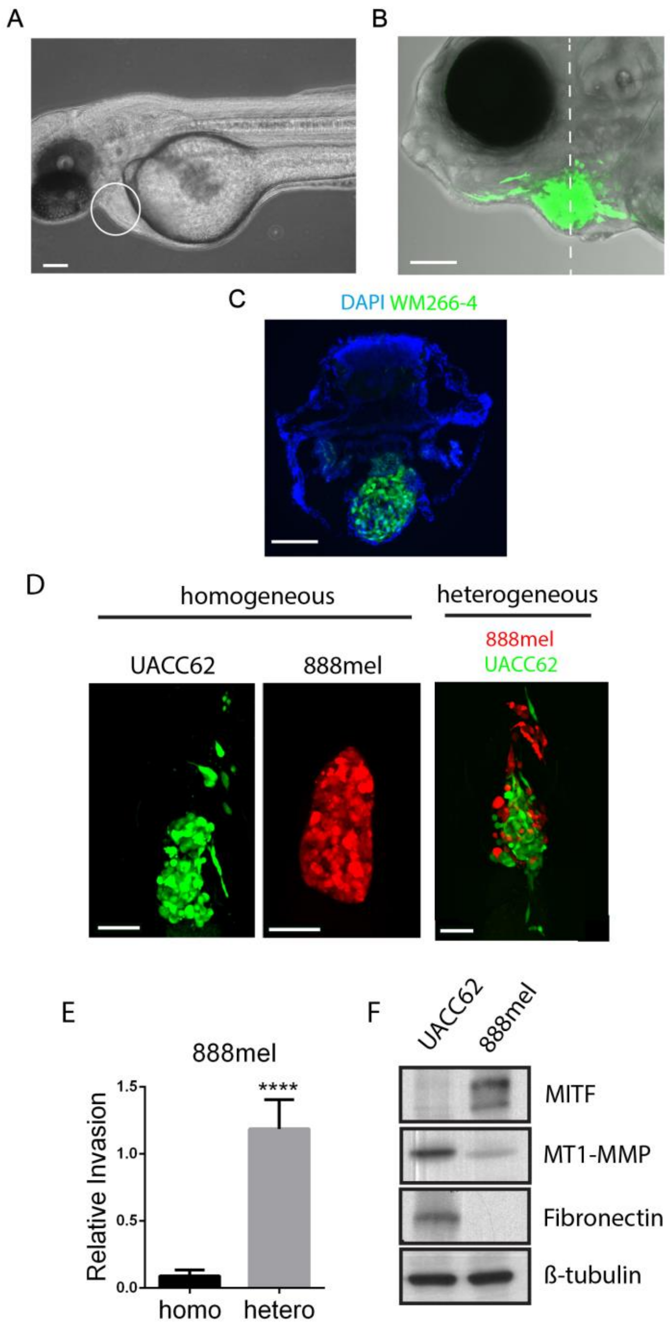

Figure S1. Zebrafish embryo melanoma xenografts. (Related to Figure 1.)

(A) A 48 hour post fertilisation zebrafish showing the pericardial cavity (white circle) as the site of xenograft injection. (B) GFP labelled WM266-4 melanoma cells injected into the pericardial cavity form a tumour-like mass capable of local invasion. Dashed line indicates plane of section in (C). (C) A cryo-section through the tumour like mass shown in (B) was labelled with DAPI (nuclei) and melanoma cells visualised through GFP fluorescence (weak autofluorescence is apparent at certain sites). The mass engrafted onto the body wall of the pericardial cavity. (D) UACC62 and 888mel homogeneous and heterogeneous xenografts. (E) Quantification of 888mel invasion depicted in (D) normalised to UACC62 invasion; mean ± SEM; Mann-Whitney test; **** = $p < 0.0001$; $N \geq 26$ from 3 independent experiments. (F) Western blot showing protein levels of MITF, MT1-MMP and fibronectin in UACC62 and 888mel cells. Scale bars = 100 μm.

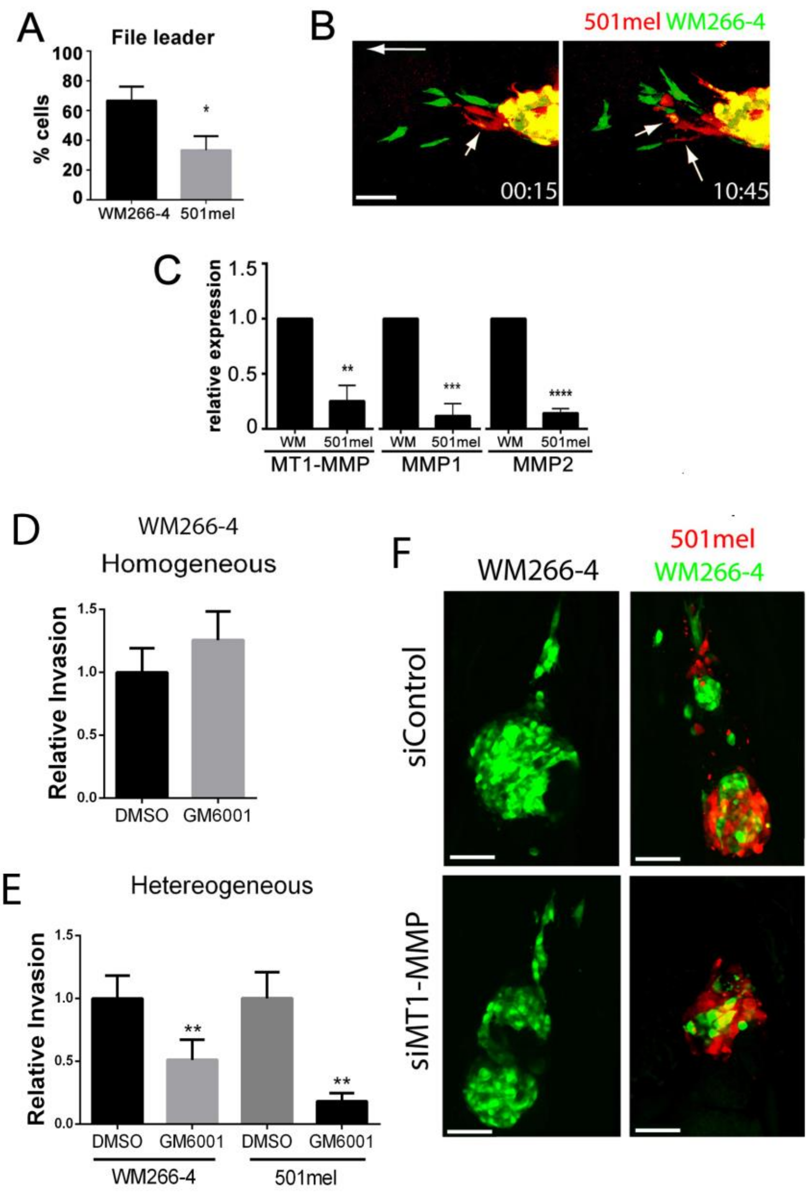

Figure S2. Follower-leader behaviour underlies co-operative invasion in heterogeneous xenografts— a crucial role for MT1-MMP. (Related to Figure 2.)

(A) Quantification of file leader; mean ± SEM; Mann-Whitney test; * = $p<0.05$; N ≥ 26 from 3 independent experiments. (B) Video time-lapse stills showing WM266-4 ‘leader’ cells and 501mel ‘follower’ cells (white arrows); scale bar = 50 μm. (C) Relative MMP expression determined by RT-qPCR from 3 independent experiments. Mean ± SEM; unpaired Student’s T-test; ** = $p <0.01$; *** = $p < 0.001$; **** = $p < 0.0001$. (D) Quantification of invasion of WM266-4 cells in homogeneous treated with either the vehicle control DMSO or GM6001; mean ± SEM; Mann-Whitney test; N ≥ 14 from 3 independent experiments. (E) Quantification of invasion of WM266-4 and 501mel cells in heterogeneous xenografts treated with either the vehicle control DMSO or GM6001; mean ± SEM; Kruskal-Wallis test followed by Dunn’s multiple comparisons test; ** = $p<0.01$; N ≥ 17 from 3 independent experiments. (F) Representative images of WM266-4 cells transfected with either control or MT1-MMP specific siRNA in homogeneous and heterogeneous xenografts.

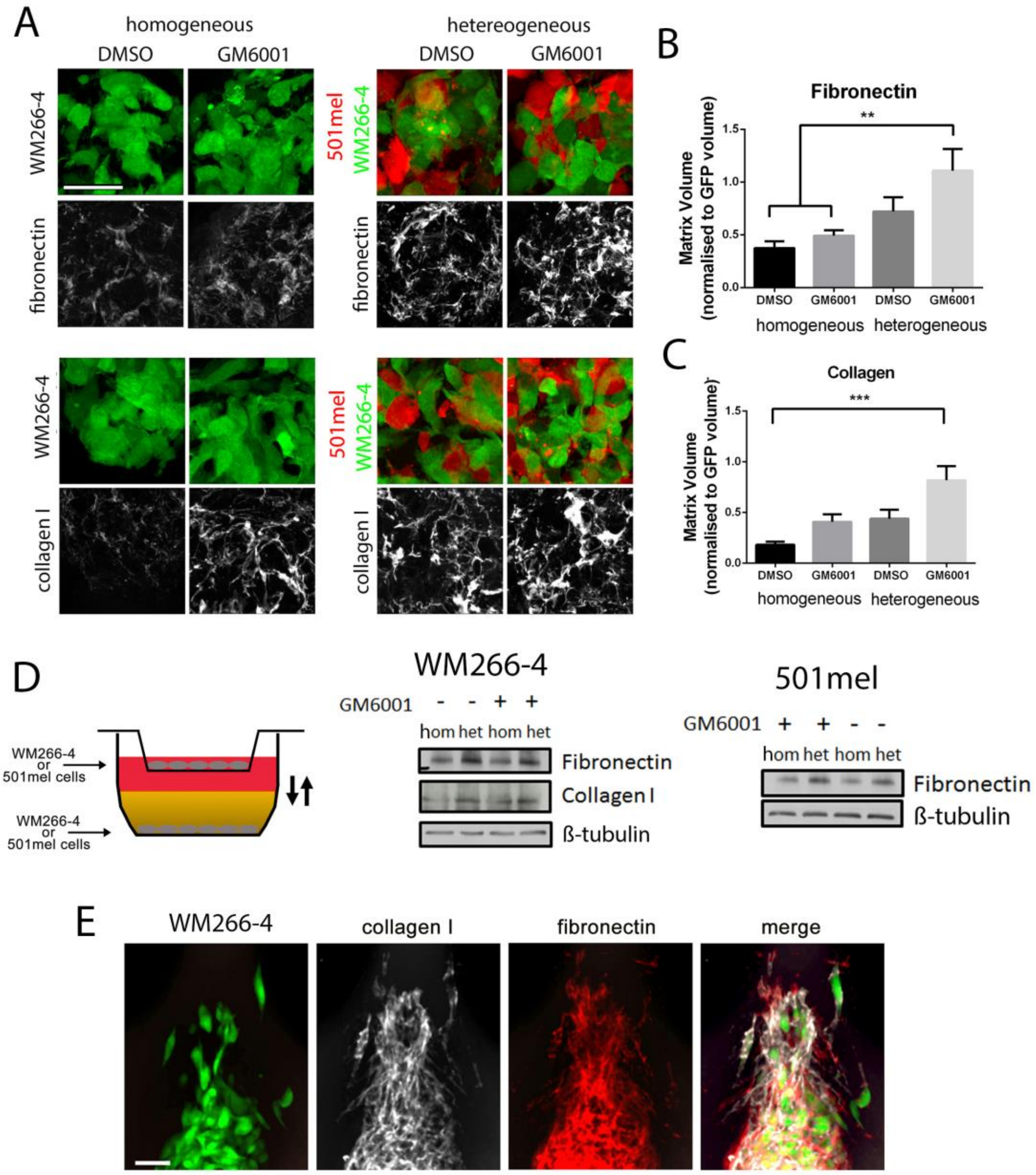

Figure S3. Characterisation of matrix deposition in xenografts. (Related to Figure 4.)

(A) Cryosections of homogeneous or heterogenous xenografts treated either with the vehicle control DMSO, or the pan-MMP inhibitor GM6001 and stained for fibronectin (upper panels) and collagen I (lower panels). (B) Quantification of fibronectin fluorescence intensity volume normalised to WM266-4 (GFP) volume of homogeneous and heterogeneous xenografts; mean ± SEM; one-way ANOVA followed by Tukey's multiple comparisons test; N ≥ 9 from 3 independent experiments. (C) Quantification of collagen I fluorescence intensity volume normalised to WM266-4 (GFP) volume of homogeneous and heterogeneous xenografts; mean ± SEM; Kruskal-Wallis followed by Dunn's multiple comparisons test N ≥ 9 from 3 independent experiments. (D) Western blot showing collagen I or fibronectin expression in either WM266-4 cells (middle panels) or 501mel (right panels) co-cultured either with autologous cells (hom) or heterologous cells (het; as depicted in the cartoon, left). Furthermore, cells were either treated with DMSO or a cocktail of protease inhibitors. (E) Wholemount immunofluorescence labelling of both fibronectin and collagen I in homogeneous WM266-4 xenografts at 4 dpi. Scale bars = 50 µm.

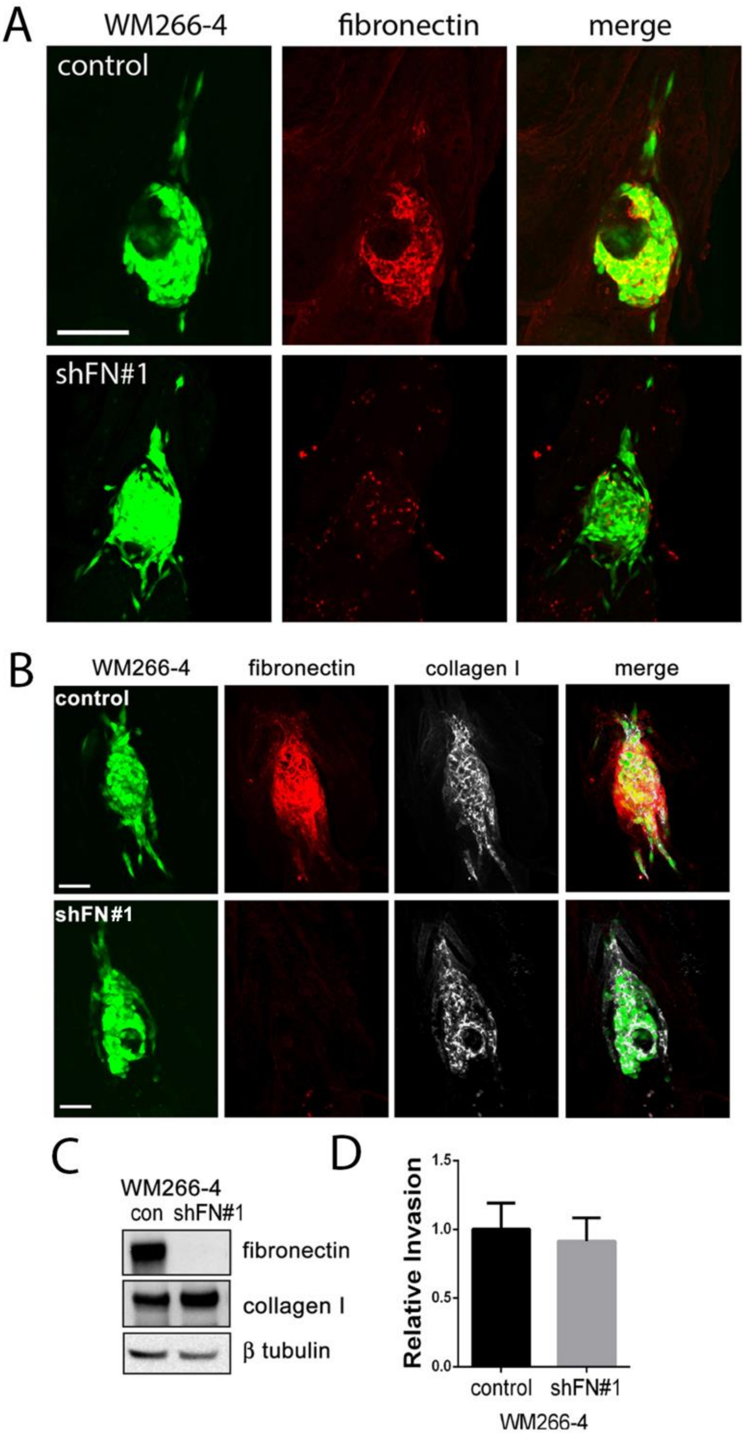

Figure S4. Fibronectin is not required for homogeneous WM226-4 cell invasion and collagen I deposition is independent of fibronectin. (Related to Figure 5.)

(A) Wholemount immunofluorescence labelling of fibronectin in homogeneous xenografts of control (upper panel) and shFN#1 WM266-4 cells (lower panel). Mean ± SEM; unpaired Student's T-test; N ≥ 15 from 3 independent experiments. (B) Wholemount immunofluorescence labelling of fibronectin and collagen I in homogeneous xenografts of control (top panel) and shFN#1 (bottom panel) WM266-4 cells. Scale bars = 100 μm. (C) Western blot showing fibronectin and collagen I expression in control and shFN#1 WM266-4 cells. (D) Relative invasion quantification of (A).

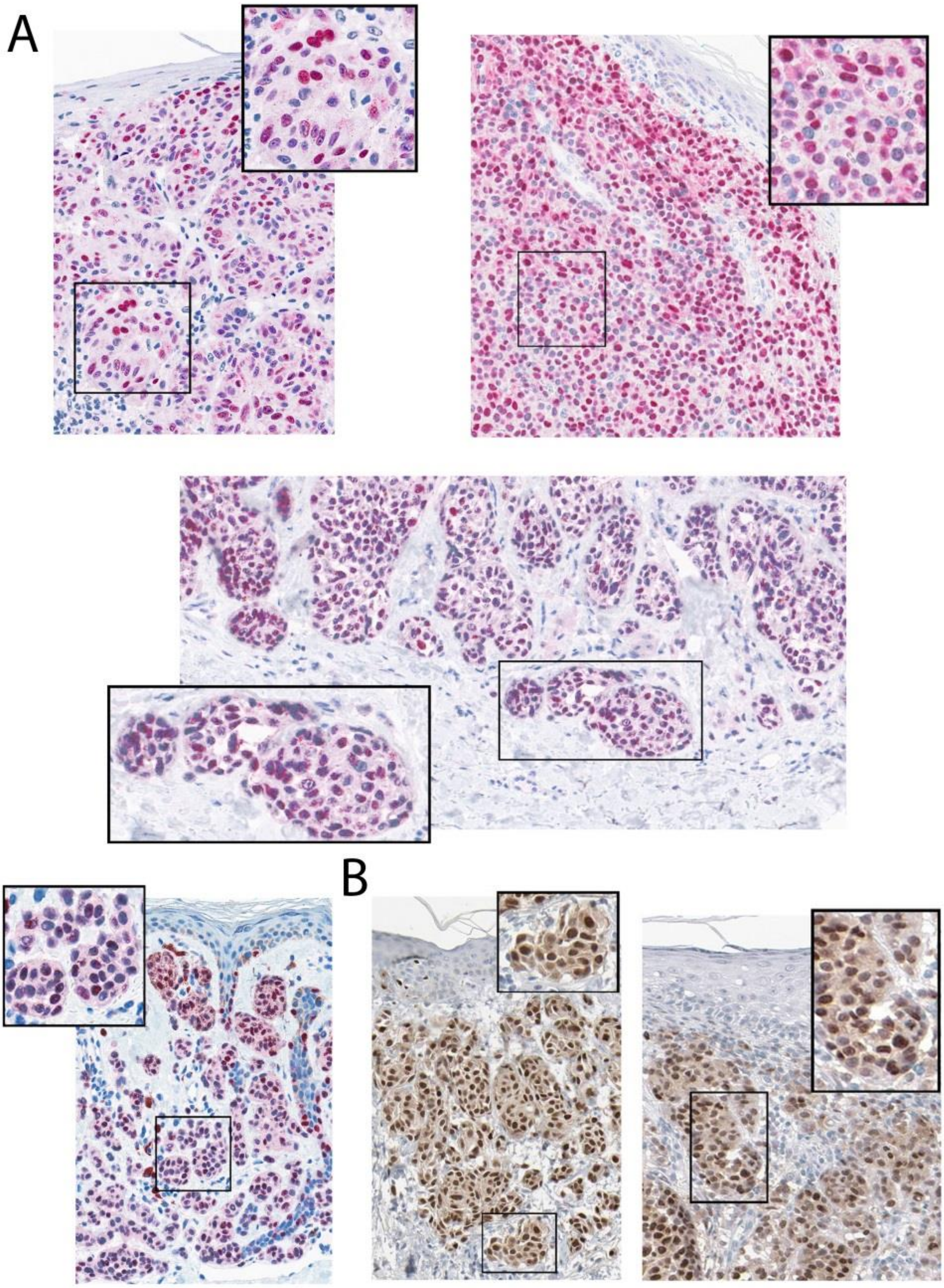

Figure S5. Invading cells are heterogeneous. (Related to Figure 5.)

MITF expression in cells during human melanoma invasion. Boxed area is magnified and reveals heterogeneous MITF expression. (B) Images courtesy of the Human Protein Atlas http://www.proteinatlas.org/ENSG00000187098/cancer/melanoma (Uhlen et al., 2010).

EXTENDED EXPERIMENTAL PROCEDURES

Cell culture

The human metastatic melanoma cell lines UACC62, WM266-4 that express GFP, 501mel and 888 mel that express mcherry were maintained at 37 ^{o}C/5 % CO_2 in DMEM (Sigma) with 10 % fetal bovine serum (Sigma) and 0.5 % penicillin-streptomycin (Sigma). 501mel and 888mel-mCherry cells were maintained in 1mg/ml G418 for selection purposes. Prior to injection, UACC62 cells were stained with 45 μM CellTrace™ CFSE Cell Proliferation Kit (Invitrogen) for 25 minutes in PBS, and washed for 30 minutes in DMEM. Knockdown of MT1-MMP in WM266-4 cells was achieved using INTERFERin (Polyplus Transfection) transfection reagent as per manufacturer's instructions and 2nM siRNA. The MT1-MMP targeted siRNA and scrambled (non-targeting) control siRNA sequences were as follows: MT1-MMP = GAUCAAGGCCAAUGUUCGA; control siRNA AAUAUAAUCACUAUCAGGUGC. Stable fibronectin knockdown (shFN) WM266-4 cells were generated with the Block-iT^{TM} Pol II miR RNAi expression vector kit (Invitrogen) using the pcDNA^{TM} 6.2-GW/EmGFP-miR vector as per manufacturer's instructions. shRNA sequences against fibronectin were as follows:
shFN#1 =
5′-TGCTGACTTCATGTTGTCTCTTCTGCGTTTTGGCCACTGACTGACGCAGAAGACAACATGAAGT-3′
and shFN#2 =
5′-TGCTGTGTACTTGGAAATGTGAGATGGTTTTGGCCACTGACTGACCATCTCACTTCCAAGTACA-3′.

The negative control provided with the kit consists of an insert that forms a hairpin structure and is processed into mature miRNA, but is predicted not to target any known vertebrate gene. WM266-4 cells were transfected with Attractene (Qiagen), cells were then selected using Blasticidin (12 μg/ml; Invitrogen), sorted for GFP expression by FACs Aria (BD Biosciences) and clones produced by limiting dilution.

Melanoma three-dimensional spheroid assay and co-culture system

5000 WM266-4-GFP cells were resuspended in DMEM containing 5% FBS and 1.5% methylcellulose (Sigma) and transferred into a 96 U-well plate for 24 hours to allow spheroid formation. Spheres were then transferred into 2 ml fibrillar bovine dermal collagen (3.1mg/ml stock, 1.9mg/ml final concentration Nucaton) containing 25 μg/ml Fibronectin from Human

Plasma (Sigma Aldrich F0895) in a 6-well plate. 501mel-mCherry or WM266-4 GFP cells plated in 6 well transwell polycarbonate membrane 24mm 0.4um inserts (Appleton Woods) were placed on top of the spheroids embedded in collagen or otherwise on top of monolayers of either 501mel-mCherry or WM266-4 GFP. Either the vehicle control DMSO or a cocktail of protease inhibitors Calpeptin (10 μM, Calbiochem), GM6001 20μM, Enzo Life Technologies), Aprotinin (10μg/ml, Sigma) Leupeptin (10μg/ml, Sigma) were added for 120 hours. Invasion of GFP expressing WM266-4 cells was monitored and images acquired using a Leica DM IL HC inverted microscope and FC340 Cooled Monocrome camera (Leica Microsystems)

RT-PCR

Cell pellets of single 501mel and WM266-4 cells were lysed with Qiazol (Qiagen). RNA was DNase treated (Qiagen) and reverse transcription performed using Omniscript (Qiagen), dNTPs (Qiagen), RNase inhibitor (Biolabs) and random hexamers (Applied Biosystems). RT-PCR was performed using SYBR Green Jumpstart (Sigma) and a Chromo4 qPCR system (BioRad) with triplicate biological repeats for each sample, and fold change calculated normalised to beta-actin expression. Primers were as follows: beta actin Fwd: GCAAGCAGGAGTATGACGAG, Rev: CAAATAAAGCCATGCCAATC, mt1mmp Fwd: AAGCAGCAGCTTCAGCCCCG Rev: GCAGCGATGGCCGCTGAGAG, mmp1 Fwd: CCAGGCCCAGGTATTGGACGGG Rev: TGGGAGAGTCCAAGAGAATGGCCG, mmp2 Fwd: ACCAGCTGGCCTAGTGATGATGT, Rev: GGGGCAGCCATAGAAGGTGTTCA.

Immunoblotting

Cell lysates were prepared using RIPA buffer (150mM NaCl; Sigma, 10mM Tris pH7.2; Sigma, 0.1% SDS, Sigma, 1% Triton-x-100, Sigma, 25mM sodium deoxychoate, Sigma, 5mM EDTA, Sigma) with added protease inhibitors (GE Healthcare). Protein concentration was determined using Pierce ® BCA protein assay kit (Thermo Scientific). Equal lysates were loaded onto a NuPage® Novex® 4-12% Bis-Tris gel (Life Technologies), proteins separated by SDS-PAGE electrophoresis and transferred to nitrocellulose membrane (ImmobilonP) according to standard protocols. Protein bands were detected using primary antibodies against: collagen I (rabbit polyclonal, 1:4000 dilution, Rockland Immunochemicals), fibronectin (rabbit polyclonal 1:5000, F3648 Sigma), beta-tubulin (rabbit polyclonal, 1:4000, Santa Cruz Biotechnologies), mitf (mouse monoclonal 1:1000 , Neomarkers) and anti ERK2 (rabbit polyclonal 1:4000m, Santa Cruz Biotechnologies). Primary antibodies were detected and visualised using an anti rabbit or anti-mouse horse radish peroxidise (HRP)-tagged secondary antibody (1:5000, GE Healthcare) and by the addition of chemi-luminescence substrate (ECL, Perkin-Elmer) and autoradiography.

Xenograft assay

Adult zebrafish (*Danio rerio*) were maintained at the University of Manchester Biological Services Unit according to National Home Office regulations under the Animals (Scientific Procedures) Act 1986. *Casper* strain (*roy*$^{-/-}$, *nacre*$^{-/-}$) zebrafish were used throughout the study to generate embryos completely lacking pigment which can otherwise obscure imaging. WM266-4 GFP, 501mel-mCherry cells, a 1:1 mix of these two cell lines, stained UACC62, 888mel-mCherry or a 2:1 mix of these two cell lines were resuspended at $1.6x10^7$cells/ml on ice with 0.5% polyvinylpyrrolidone K60 solution (PVP, Sigma). 48 hours post fertilisation (hpf) embryos were anaesthetised with 0.1mg/ml MS222 (Sigma) and approximately 500 cells were injected into the pericardial cavity, using a micropipette and pump (World Precision Instruments). Engrafted embryos were maintained at 34°C for 4 days. As needed, embryos were treated at 1 day post injection (dpi) with the vehicle control 0.1% dimethyl sulfoxide (DMSO, Sigma) or a cocktail of protease inhibitors: Calpeptin (1.5μM, Calbiochem), GM6001 (3μM, Enzo Life Technologies), Aprotinin (1.5μg/ml, Sigma) Leupeptin (1.5μg/ml, Sigma) or GM6001 alone (20μM, Sigma) for 72 hours. At 4dpi, embryos were fixed in 4% parafomaldehyde (PFA, Sigma) in PBS at 4°C overnight.

Wholemount immunofluorescence

Fixed 4 dpi zebrafish embryos were washed in PBS, acetone cracked in ice-cold acetone for 10 minutes on ice and blocked for 1 hour in PBDT buffer (PBS with 1 % DMSO, 1 % bovine serum albumin (BSA; Sigma), 0.5 % triton X-100 (Sigma)) and 5 % goat serum (Sigma). Zebrafish were incubated overnight with anti-collagen I (1:800 dilution, rabbit polyclonal; Rockland Immunochemicals) and anti-fibronectin (1:1000 dilution, Ab6328; Abcam) at 4 ^{o}C in PBDT buffer. Zebrafish were washed in PBS with 0.5 % Triton-x-100 (PBST) and incubated with secondary Alexa Fluor antibodies: anti-mouse 594, anti-mouse 647, anti-rabbit 594 and anti-rabbit 647 (1:150 dilution, Invitrogen) for 2 hours in PBDT followed by washes with PBST.

Sectioning

Fixed 4 dpi zebrafish embryos were either cryo-preserved in 30 % sucrose (Sigma) in water overnight before equilibration in OCT for 1 hour, or cryo-preserved in 20% sucrose for 3 hours before equilibration in 15% fish gelatin (Sigma) overnight. Embedded zebrafish embryos were frozen and 12 μm sections were cut using a cryostat (Leica model). Sections were warmed, permeabilised in 1% saponin, incubated overnight with anti-MITF antibody (1:40, Leica), anti-collagen antibody (as before) or anti-fibronectin antibody (as before) and incubated with Alexa-

Fluor anti-mouse 647 as before. Sections were mounted in vectashield with DAPI (VectaLabs) for imaging nuclei.

Microscopy

1 dpi engrafted zebrafish embryos were anaesthetised in 0.1mg/ml MS222. Fixed 4 dpi engrafted zebrafish were mounted in 1.5 % low melting agarose (LMP, Flowgen Biosciences). Tumours were imaged using a Leica TCS SP5 AOBS upright confocal (Leica Microsystems) using a 20 x 0.50 Plan Fluotar dipping objective and 1.5 x confocal zoom. When it was not possible to eliminate cross-talk between channels, the images were collected sequentially. Z stacks from the top to the bottom of the tumour were captured and the maximum intensity projections of these 3D stacks are shown in the results. Zebrafish embryos sections were imaged using Leica TCS SP5 AOBS inverted confocal using a 40 x 0.50 Plan Fluotar objective and 2 x confocal zoom. Z stacks were captured and the maximum intensity projections are shown.

MITF Immunohistochemistry

To check the expression of MITF in human melanoma biopsies, formalin fixed, paraffin embedded archive samples were stained by immunohistochemistry after antigen recovery. Briefly after deparaffinisation, antigen retrieval was performed by the pressure cooker method using EDTA buffer, pH 8.0. Staining was performed using an automated system (Autostainer plus Dako). A monoclonal mouse antibody raised against a N-terminal fragment of MITF protein of human origin (Santa Cruz Biotechnology, sc-56433) was used at a dilution of 1:60.

Analysis

Captured z stacks were processed using Volocity software (Perkin Elmer, Cambridge, UK). All experiments consist of a minimum of three independent repeats. Relative Invasion Index (RII) is defined as the average number of cells invaded outside the pericardial cavity at 4dpi normalized to the average number in a control group. All data sets were tested for normality using D'Agostino & Pearson omnibus normality test. All statistics were based on continuous variables. Comparisons of two data sets were performed using the paired Student's T-Test (parametric) or Mann-Whitney test (non-parametric). Comparisons of more than two data sets were performed using one-way ANOVA followed by Tukey's multiple comparison test (parametric) or Kruskal-Wallis test followed by Dunn's multiple comparison test (non-parametric). Appropriate statistical tests are annotated in figure legends. All statistical analysis was performed using GraphPad Prism version 6 (GraphPad Software Inc).
