# Supplementary material for: Heterogeneous Tumor Subpopulations Cooperate to Drive Invasion
Source: Cell Rep. 2014 Jul 24;8(3):688–95. doi: 10.1016/j.celrep.2014.06.045 (PMC4542310; doi:10.1016/j.celrep.2014.06.045)
Supplement: Document S2. Article plus Supplemental Information [file mmc2.pdf]

OPEN ACCESS
CellPress

Cell Reports
Report

Heterogeneous Tumor Subpopulations Cooperate to Drive Invasion

Anna Chapman,[1] Laura Fernandez del Ama,[1] Jennifer Ferguson,[1] Jivko Kamarashev,[2] Claudia Wellbrock,[1,3,*] and Adam Hurlstone[1,3,*]
[1]Faculty of Life Sciences, The University of Manchester, Oxford Road, Manchester, M13 9PT, UK
[2]Department of Dermatology, University Hospital Zürich, Gloriastrasse 31, 8091 Zürich, Switzerland
[3]Co-senior author
*Correspondence: claudia.wellbrock@manchester.ac.uk (C.W.), adam.hurlstone@manchester.ac.uk (A.H.)
http://dx.doi.org/10.1016/j.celrep.2014.06.045
This is an open access article under the CC BY license (http://creativecommons.org/licenses/by/3.0/).

SUMMARY

Clonal selection and transcriptional reprogramming (e.g., epithelial-mesenchymal transition or phenotype switching) are the predominant theories thought to underlie tumor progression. However, a "division of labor" leading to cooperation among tumor-cell subpopulations could be an additional catalyst of progression. Using a zebrafish-melanoma xenograft model, we found that in a heterogeneous setting, inherently invasive cells, which possess protease activity and deposit extracellular matrix (ECM), co-invade with subpopulations of poorly invasive cells, a phenomenon we term "cooperative invasion". Whereas the poorly invasive cells benefit from heterogeneity, the invasive cells switch from protease-independent to an MT1-MMP-dependent mode of invasion. We did not observe changes in expression of the melanoma phenotype determinant MITF during cooperative invasion, thus ruling out the necessity for phenotype switching for invasion. Altogether, our data suggest that cooperation can drive melanoma progression without the need for clonal selection or phenotype switching and can account for the preservation of heterogeneity seen throughout tumor progression.

INTRODUCTION

Tumors comprise subpopulations of transformed cells that are genotypically or phenotypically divergent. In melanoma, cell subpopulations have been characterized that differ in gene expression profiles, proliferation rates, and invasiveness; leading to the definition of so-called proliferative and invasive phenotypes that correlate with relatively high and low expression, respectively, of the melanocyte lineage determinant MITF (Hoek et al., 2006, 2008). However, whether interactions between heterogeneous melanoma cell subpopulations contribute to invasion and metastasis is unknown. To study the potential significance of heterogeneity for the dissemination of melanoma cells, we developed a xenograft model in zebrafish embryos that allows monitoring of local invasion with high resolution. To represent genotypes relevant to melanoma we selected melanoma cell line pairs that harbor the V600E BRAF mutation, the most common mutation present in melanoma (Wellbrock and Hurlstone, 2010), but differ in their expression of MITF, and as a consequence the individual cell lines are either inherently invasive (MITF^{low}) or poorly invasive (MITF^{high}). We find that these divergent cell lines communicate reciprocally and cooperate to invade collectively dependent on protease activity and fibronectin deposition and without altering MITF expression.

RESULTS

Heterogeneity Confers Invasive Properties on Individually Poorly Invasive Melanoma Cells

Invasive MITF^{low} WM266-4 cells or poorly invasive MITF^{high} 501mel cells (Figure 1A; Arozarena et al., 2011; Ohanna et al., 2011; Rozenberg et al., 2010) were injected into the pericardial cavity of zebrafish embryos at 48 hr postfertilization (Figures S1A and S1B available online). Both melanoma cell lines (WM266-4-GFP and 501mel-mCherry) aggregated rapidly and anchored to the body wall to form tumor-like masses (Figures 1B and S1C). As anticipated, WM266-4 cells invaded efficiently, but 501mel cells displayed little invasion (Figure 1B). Strikingly, however, in a heterogeneous situation, when WM266-4 and 501mel cells were present in equal ratios within the xenograft, the invasion of 501mel cells increased markedly (Figure 1C). In tissue sections of engrafted zebrafish embryos, infiltrating tumor cells were found migrating away from the primary site through solid tissue (Figure 1D). We enumerated the invading cells located outside the pericardium (dashed line in Figure 1D). This confirmed that the invasion of 501mel cells increased to levels similar to WM266-4 cells (Figure 1E). This striking behavior was also observed for another pair of MITF^{low} and MITF^{high} melanoma cell lines—UACC62 and 888mel cells, respectively—(Figures S1D–S1F), suggesting this may be a general phenomenon. Thus, melanoma cells that display divergent invasive phenotypes in isolation interact symbiotically under heterogeneous circumstances, a phenomenon that we describe as "cooperative invasion".

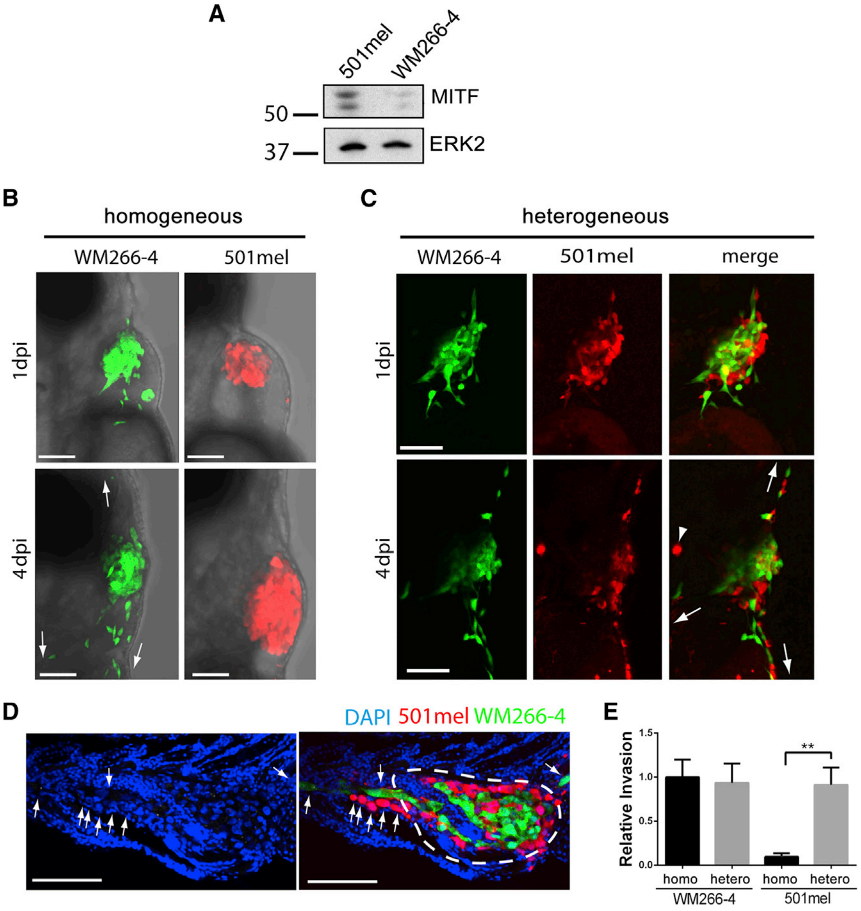

Figure 1. Heterogeneity Results in Cooperative Invasion

(A) Western blot showing MITF expression in WM266-4 and 501mel cells.

(B) Homogeneous xenografts imaged at 1 (upper) and 4 days (lower) postinjection (dpi).

(C) Heterogeneous xenografts imaged at 1 (upper) and 4 dpi (lower).

Arrows indicate directions of invasion; arrowhead indicates autofluorescence.

(D) Section from engrafted embryo indicating primary tumor site (white dashed line) and infiltrating melanoma cells (white arrows). Scale bars represent 100 μm.

(E) Quantitation of invasion depicted in (A) and (B); mean ± SEM; Kruskal-Wallis test followed by Dunn's multiple comparisons test; **$p < 0.01$; $n \geq 26$ from three independent experiments.

See also Figure S1.

Cooperative Invasion Requires the Activity of MT1-MMP

Further analysis revealed that WM266-4 cells were significantly more likely to be the leading cell of an invasive file (Figure S2A). Live imaging confirmed that over time WM266-4 cells lead files of invading cells (Figure S2B). Such behavior has been described for tumor-associated fibroblasts, which contribute to the matrix metalloproteinase (MMP)-mediated degradation of the extracellular membrane (ECM), thereby enabling epithelial cancer cells to invade (Gaggioli et al., 2007). Real-time PCR revealed significantly higher expression of the three most prominent cancer related MMPs—MMP1, MMP2 and MT1-MMP—in WM266-4 cells compared to 501mel cells (Figure S2C), suggesting that during cooperative invasion, WM266-4 cells could adopt a role similar to fibroblasts.

We assessed the relevance of protease activity for cooperative invasion by incubating engrafted embryos with a previously described cocktail of protease inhibitors (Sahai and Marshall, 2003; Wolf et al., 2003). This had no effect on homogeneous 501mel xenografts, where no invasion occurred in any case (Figures 2A and 2B). However, the invasion of 501mel cells in heterogeneous xenografts was almost completely blocked in the presence of the inhibitor mix (Figures 2A and 2B). This indicates that the proteolytic cleavage of ECM is necessary for the acquired invasion of 501mel cells. Homogeneous WM266-4 xenografts showed no difference in relative invasion when treated with the protease inhibitors compared to DMSO (Figures 2A and 2C), suggesting that WM266-4 cells can use a proteolytic-independent mechanism of invasion, as has been described elsewhere (Sahai and Marshall, 2003; Wolf et al., 2003). Surprisingly, however, in heterogeneous xenografts treated with the protease inhibitor mix WM266-4 cells showed a dramatic decrease in invasion (Figures 2A and 2C). Suppression of cooperative invasion but not invasion of homogeneous WM266-4 cells was also observed when the pan-MMP inhibitor GM6001 was used alone, albeit at higher concentration (Figures S2D and S2E). Because MT1-MMP is a major regulator of protease-driven invasion (Sabeh et al., 2004), we depleted MT1-MMP expression in WM266-4 cells using RNAi; this efficiently suppressed cooperative invasion, although again did not affect the invasion of homogeneous WM266-4 cells (Figures 2D–2F and S2F). Further corroborating the importance of MT1-MMP for cooperative invasion, invasive UACC62 cells also express significantly more MT1-MMP than poorly invasive 888mel cells (Figure S1F). Thus, we not only identify a crucial role for MT1-MMP in cooperative invasion, but also demonstrate that it is tumor cell protease activity rather than host protease activity that is required for cooperative invasion.

The preceding experiments indicated that the presence of 501mel cells suppressed the protease-independent invasive potential of WM266-4 cells. To further investigate the mechanism of crosstalk, we cultured WM266-4 cells as spheroids embedded in pepsin-extracted bovine collagen in the absence or presence of 501mel cells and added protease inhibitors to the culture system (Figure 3A). We found that under these conditions, homogeneous WM266-4 cells invaded the matrix singly with a predominantly rounded morphology (Figure 3B), in line with previously published data (Arozarena et al., 2011; Sahai and Marshall, 2003). However, exposure to soluble factors

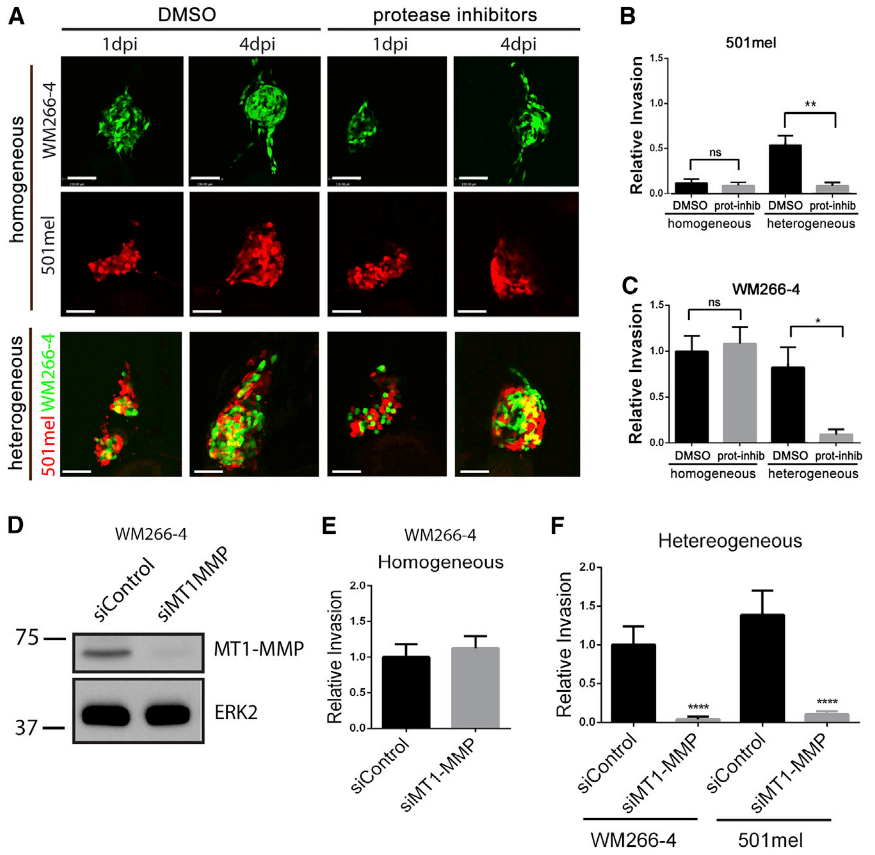

Figure 2. MMP Inhibition Suppresses Cooperative Invasion

(A) Homogeneous (upper) or heterogeneous (bottom) xenografts were treated with either the vehicle control DMSO (left) or protease inhibitor cocktail (right). Scale bars represent 100 μm.

(B) Quantitation of 501mel invasion depicted in (A); mean $\pm$ SEM; Kruskal-Wallis test followed by Dunn's multiple comparisons test; $^{**}p < 0.01$; $n \geq 9$ from three independent experiments.

(C) Quantitation of WM266-4 invasion depicted in (A); mean $\pm$ SEM; Kruskal-Wallis test followed by Dunn's multiple comparisons test; $^{*}p < 0.05$; $n \geq 13$ from three independent experiments.

(D) Western blot showing MT1-MMP expression in WM266-4 transfected with either control or MT1-MMP specific siRNA.

(E) Quantitation of invasion of WM266-4 cells in homogeneous xenografts wherein WM266-4 cells have been transfected with either control or MT1-MMP-specific siRNA; mean $\pm$ SEM; Mann-Whitney test; $n \geq 21$ from three independent experiments.

(F) Quantitation of invasion of WM266-4 and 501mel in heterogeneous xenografts wherein WM266-4 cells have been transfected with either control or MT1-MMP specific siRNA; mean $\pm$ SEM; Kruskal-Wallis test followed by Dunn's multiple comparisons test; $^{****}p < 0.0001$; $n \geq 24$ from three independent experiments.

See also Figure S2.

derived from 501mel cells resulted in a reduction of invasion and a switch to an elongated mode of invasion (Figure 3B), which is known to be protease dependent.

Cooperative Invasion Involves Changes in the ECM

MMPs and fibronectin are coexpressed in melanoma cells (Kamoshida et al., 2013), supporting the idea that ECM deposition and degradation are closely coordinated. Also, fibronectin is associated with melanoma progression (Gaggioli et al., 2005) and can regulate the organization of type I collagen fibrils (Sottile and Hocking, 2002). To analyze the involvement of type I collagen and fibronectin in cooperative invasion, we first performed whole-mount immunofluorescence staining on homogeneous WM266-4 and 501mel xenografts: WM266-4 xenografts were surrounded by abundant type I collagen and fibronectin, but this was not detectable in 501mel xenografts (Figure 4A). Western blotting revealed a strong expression of both ECM proteins in WM266-4 cells, but barely detectable expression in 501mel cells (Figure 4B), suggesting that the observed ECM in the xenografts is derived from WM266-4 cells.

Intriguingly, ECM detected in heterogeneous xenografts was more abundant than in WM266-4 homogeneous xenografts (Figures 4C and S3A–S3C), a further indication of reciprocal communication between the two subpopulations. Additionally, coculturing WM266-4 cells with 501mel cells in a transwell system resulted in increased ECM protein expression in both cell lines (Figure S3D), suggesting the involvement of diffusible factors in this communication. Moreover, ECM deposition was further augmented in xenografts comprising WM2664-cells depleted for MT1-MMP or treated with protease inhibitors, and matrix components were more diffuse (Figures 4C and S3A–S3C). However, protease inhibition did not lead to further collagen I and fibronectin induction in our in vitro coculture system (Figure S3D). This implies that the increased amount and disorder observed because of protease inhibition in vivo (Figures 4C and S3A–S3C) may be due to modulating ECM turnover rather than expression.

Cooperative Invasion Depends on Fibronectin

In addition to the ECM around the WM266-4 tumor mass, we also observed ECM around invading cells (Figure 4A). Close examination revealed fibers of fibronectin and collagen I radiating away from the xenograft (Figure S3E), typical of the arrays of collagen fibers detected around breast tumors that are associated with files of invading cells (Provenzano et al., 2006). In heterogeneous xenografts, we observed that both WM266-4 and 501mel cells appear to migrate in close connection to the tracks of ECM (Figure 4C). Quantitation showed that during cooperative invasion, cells were found predominantly on these collagen and fibronectin tracks (Figure 4D).

To test whether ECM deposition was essential for cooperative invasion, we generated WM266-4 cells expressing low levels of fibronectin through RNA interference (WM266-4 shFN#1 and shFN#2 cells). The reduction of fibronectin production was confirmed in vitro with western blotting (Figure 5A). In vivo, the

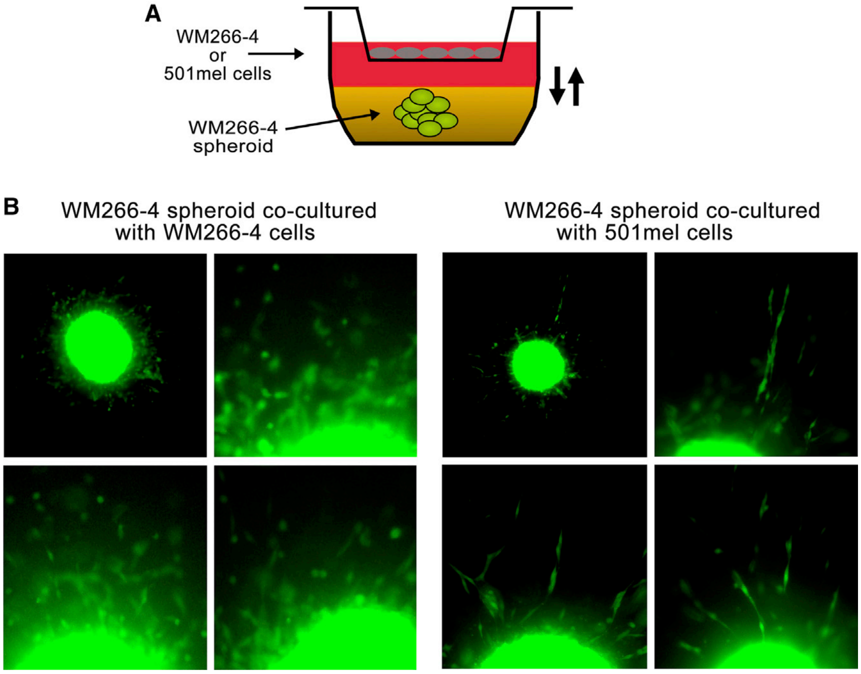

Figure 3. A Diffusible Factor Emanating from 501mel Cells Modulates WM266-4 Cell Response to Protease Inhibitors

(A) Cartoon depicting experimental set-up, with WM266-4 spheroids being cocultured either with autologous cells or heterologous cells in porous transwells.

(B) Representative images of WM266-4 spheroids cocultured either with WM266-4 or 501mel cells in the presence of a cocktail of protease inhibitors.

presence of WM266-4 shFN cells led to a significant loss of fibronectin deposition around either homogeneous or heterogeneous xenografts when compared to xenografts containing control WM266-4 cells (Figures 5B and S4A), confirming that the fibronectin associated with the xenograft was derived largely from WM266-4 cells. When we analyzed collagen deposition, we found that fibronectin knockdown did not affect the expression of collagen I in WM266-4 or its deposition in vivo (Figures S4B and S4C), suggesting that collagen deposition was independent of fibronectin. When WM266-4 shFN cells were injected as a heterogeneous mixture with 501mel cells, the invasion of 501mel cells was dramatically reduced when compared to heterogeneous xenografts containing WM266-4 control cells (Figures 5B and 5C), and more so for shFN#1 than shFN#2 cells reflecting the magnitude of fibronectin knockdown. This suggested that cooperative invasion of 501mel cells requires the presence of WM266-4 generated fibronectin tracks. In line with such a role for fibronectin, invasive UACC62 cells also express significantly more fibronectin than 888mel cells (Figure S1F). Interestingly, the reduction in fibronectin expression appeared to not affect the invasion of WM266-4 cells in homogeneous xenografts (Figure S4D). However, their invasion was impaired in heterogeneous tumors when fibronectin deposition was suppressed (Figures 5B and 5C), further supporting a reciprocal communication between the individual melanoma cell subpopulations in a heterogeneous setting.

MITF^{high} and MITF^{low} Cells Are Present in the Invasive Front of Tumors

One of the major determinants of melanoma heterogeneity is the regulation of MITF expression by the tumor microenvironment (Hoek et al., 2008). Further, a microenvironment-induced switch to a MITF^{low} phenotype is thought to drive tumor invasion (Carreira et al., 2006). Although MITF expression correlates with the invasiveness of individual cell populations (see Figure 1B and Figure S1D), the cooperative invasion observed in heterogeneous xenografts (see Figures 1C and S1D) suggested that both MITF^{high} and MITF^{low} cells could contribute to tumor invasion. Indeed, immunofluorescence to detect MITF expression in heterogeneous xenografts revealed that MITF^{high} and MITF^{low} cells invade together (Figure 5D), indicating that MITF downregulation is not required for cooperative invasion. To extend these findings into the clinical setting, human melanoma biopsies were also examined for MITF expression. Consistent with MITF heterogeneity being present in invading cells, MITF staining revealed that MITF^{high} and MITF^{low} cells coexist in groups of melanoma cells invading the dermis (Figure S5A), which is also apparent in other published data (King et al., 1999) and in biopsy samples displayed in the Human Protein Atlas http://www.proteinatlas.org (Uhlen et al., 2010; Figure S5B).

DISCUSSION

Tumors usually display a high degree of genotypic and phenotypic heterogeneity, but the impact of heterogeneity on tumor progression is not understood. Using a novel xenograft model, we explored the possibility of phenotypically divergent melanoma cells cooperating during the first steps of tumor progression by analyzing their invasive behavior. We describe here what we call cooperative invasion, during which heterogeneous melanoma cell subpopulations interact reciprocally and mobilize collectively.

Our data suggest that both proteolytic activity as well as ECM deposition are necessary for cooperative invasion because disruption of either completely abrogates invasion in heterogeneous xenografts. Moreover, we show that protease activity is required to organize rather than simply degrade the ECM. It is known that the ECM within a tumor is distinct from normal tissue, due not only to alterations in composition, but also through increased ECM stiffness (Levental et al., 2009). Alterations in tension alone can be sufficient to alter tumor cell invasion via integrin activation (Friedland et al., 2009). Thus, WM266-4-produced fibronectin could trigger changes in integrin signaling in 501mel cells, or provide tracks serving as paths for invading 501mel

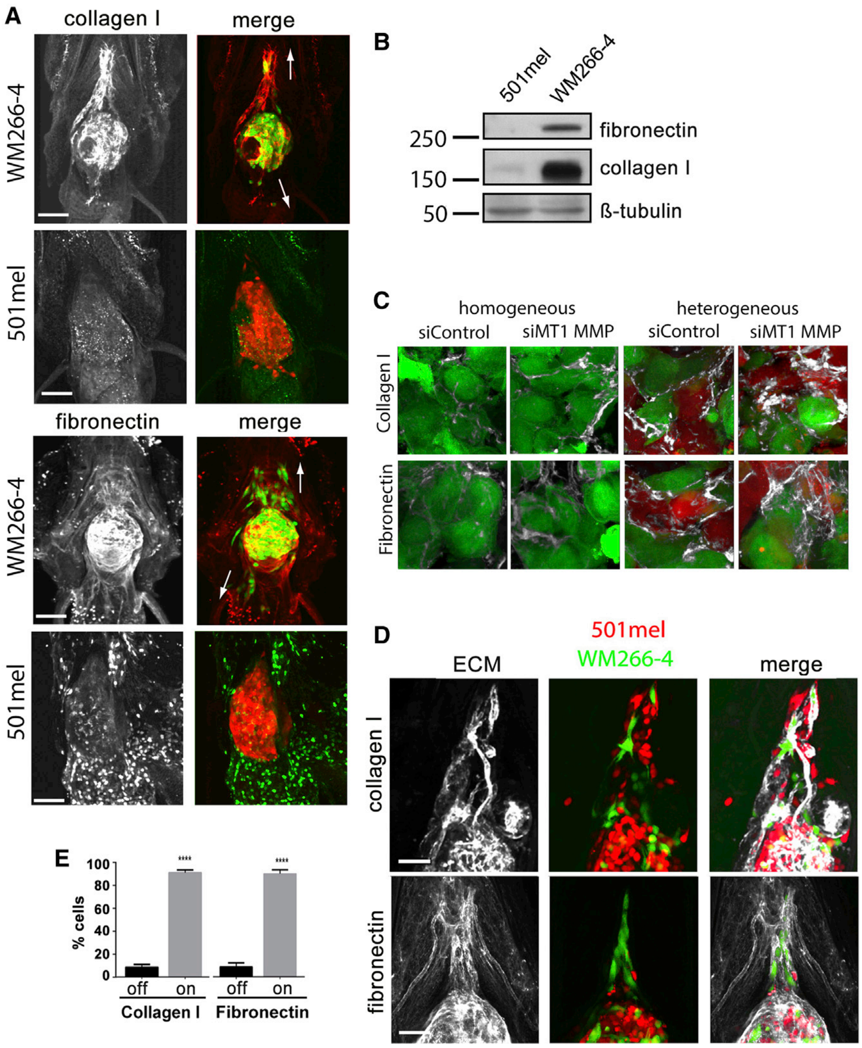

Figure 4. ECM Proteins Correlate with Invasiveness

(A) Expression of ECM components collagen I and fibronectin in engrafted zebrafish 4 dpi; arrows indicate direction of invasion.

(B) Western blot showing collagen I and fibronectin expression in WM266-4 and 501mel cells.

(C) Collagen I (upper) and fibronectin (lower) in homogeneous compared to heterogeneous xenografts that are further treated with either DMSO or GM6001.

(D) Invasive WM266-4 and 501mel follow collagen I (upper) and fibronectin (lower) tracks radiating out from the tumor.

(E) Quantitation of ECM association. Cells were scored as being in touch "on" with collagen I or fibronectin strands or not "off." Mean ± SEM; unpaired Student's t test (collagen) and Mann-Whitney test (fibronectin); ****$p < 0.0001$; $n \geq 18$ from three independent experiments.

Scale bars represent 100 μm. See also Figure S3.

cells. However, the full mechanism of this role of fibronectin is yet to be resolved.

Another important finding from our study is that the mode of invasion of WM266-4 cells switches from protease independent in homogeneous xenografts to MT1-MMP dependent in heterogeneous xenografts and resembles what has been described as collective invasion of chains of invading cells (Wolf et al., 2007). We hypothesize that 501mel cells secrete factors that induce an elongated morphology in WM266-4 cells thereby constraining them to adopt a protease-driven leader role in invasion; however, this soluble "switch factor" can be cleaved by proteases secreted by WM266-4 cells, allowing them to use "rounded" invasion when the "switch factor" is sufficiently neutralized. Potentially the same or possibly alternative secreted factors emanating from 501mel cells also augment ECM density in heterogeneous xenografts, which would too promote a protease-dependent invasion mode (Wolf et al., 2013). Together, our data highlight that when cells cooperate, the underlying cell-cell communications produce reciprocal effects on the individual subpopulations (see Figure 5E for a model). Identifying the underlying mechanisms will be crucial if we are to fully understand the impact of heterogeneity on invasion. Moreover, the reciprocal communication underlying cooperation may itself be a therapeutic target.

Tumor progression is a complex cascade of events requiring tumor cells to detach from the primary tumor, invade locally, intravasate, survive in circulation, extravasate, and finally colonize distant organs. How cancer cells acquire all these capabilities has been the subject of considerable speculation. Historically, competition between genetically divergent cancer cell variants leading to expansion of the "fittest" clone was thought to drive metastasis (Greaves and Maley, 2012). However, in multiple tumor types, secondary tumors retain the heterogeneity of primary tumors, display remarkably similar gene expression, and have very similar constellations of mutations (Ramaswamy et al., 2003; Vakiani et al., 2012; Vignot et al., 2013; Walter et al., 2012), challenging the clonal expansion model.

An alternative theory proposes that cancer cells switch behavior reversibly in response to transient changes in gene expression, which are triggered by microenvironmental cues (e.g., hypoxia or inflammation). As such, in carcinomas, reversible transitions between epithelial and mesenchymal phenotypes (EMT ↔ MET) throughout tumor progression might explain some of the similarity seen in primary and secondary tumors (Scheel and Weinberg, 2012). In melanoma, phenotype switching is thought to follow altered MITF expression (Hoek and Goding, 2010). In both examples, a switch is proposed to occur to generate different phenotypes with only one phenotype being compatible with a particular stage of tumor progression. However, this is contradicted by our observation that the invasive front in both our xenograft model and patient biopsies comprise

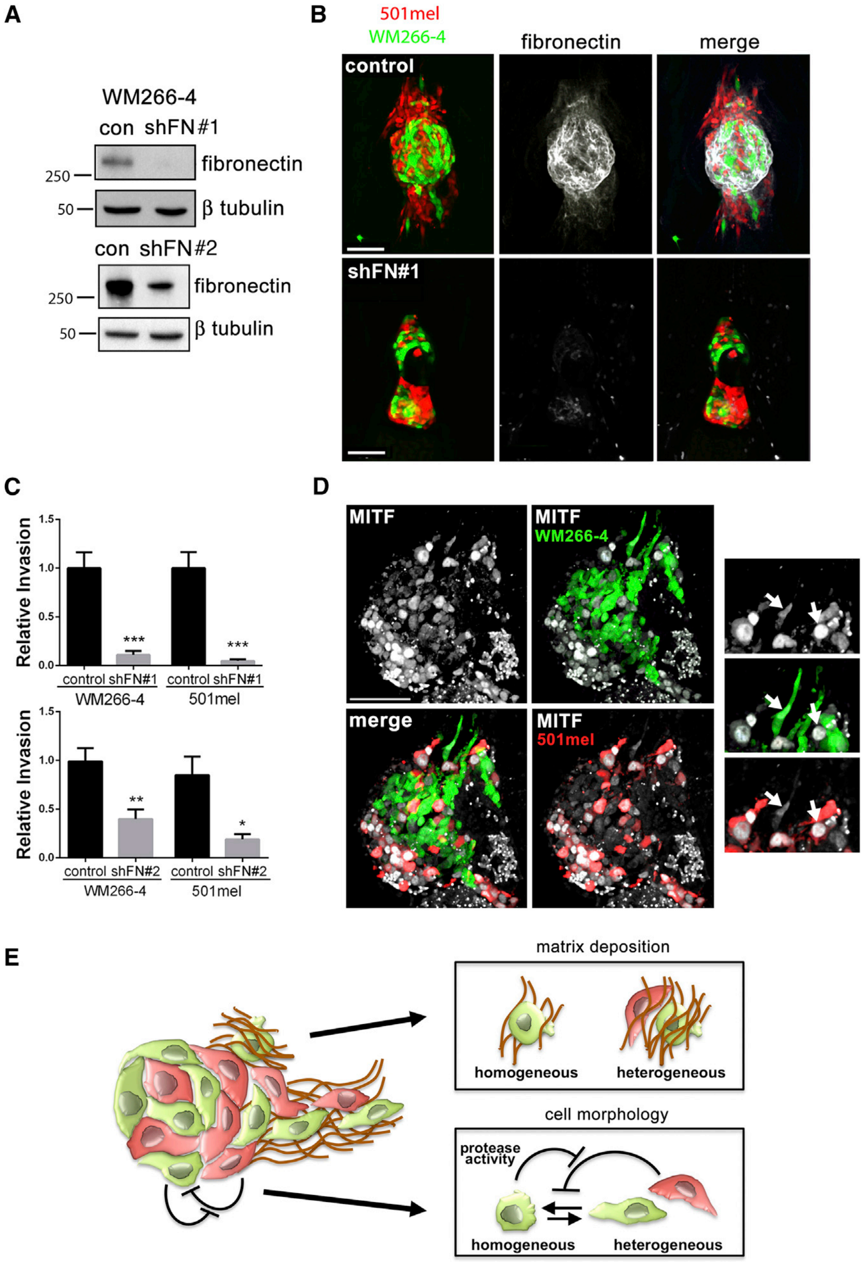

Figure 5. Fibronectin Is Essential for Cooperative Invasion; Invasive Primary Melanoma Cells Are Also Heterogeneous

(A) Western blot showing stable knockdown of fibronectin in WM266-4 GFP shFN cells. #1 and #2 are clones expressing independent shRNA targeting fibronectin. Control (con) cells express an irrelevant shRNA.

(B) Fibronectin associated with heterogeneous xenografts comprising either control WM266-4 cells (upper) or WM266-4 shFN#1 cells (lower).

(C) Quantitation of invasion of 501mel and WM266-4 cells from heterogeneous xenografts comprising either control WM266-4 cells, WM266-4 shFN#1, or WM266-4 shFN#2 cells. Mean ± SEM; Mann-Whitney test; $*p < 0.05$, $**p < 0.001$, $***p < 0.001$; $n \geq 18$ from three independent experiments.

(D) MITF immunofluorescence in frozen sections of heterogeneous xenografts. Arrows indicate high and low MITF fluorescence intensity in invading cells.

(E) Model depicting the reciprocal interactions underlying cooperative invasion.

See also Figures S4 and S5.

heterogeneous cells. Moreover, melanoma cell clusters circulating in patient blood were shown to express MITF heterogeneously (Khoja et al., 2014).

We believe that a process that maintains tumor heterogeneity throughout progression based on cancer cell cooperation better explains the preservation of heterogeneity in metastases. In support of this notion, other examples have emerged that demonstrate cooperative behavior among cancer cells. For instance, in *Drosophila*, separate clones of cells bearing RasV12 and *scribble* mutations can interact to propagate Jnk signaling resulting in neoplasia (Wu et al., 2010). In a mouse breast cancer model driven by MMTV-Wnt1, tumors were identified containing distinct basal *Hras*^{mut} *Wnt1*^{low} and luminal *Hras*^{wt} *Wnt1*^{high} subclones, both of which were required for efficient tumor propagation (Cleary et al., 2014). Furthermore, carcinoma cells that had undergone EMT can cooperate with cancer cells that still possess all epithelial traits to induce lung metastasis (Tsuji et al., 2008), and EMT and non-EMT cells were also discovered coexisting in circulating tumor cell clusters (Hou et al., 2012; Yu et al., 2013). Symbiotic interactions between individual cancer cell subpopulations may well affect drug responses (as we have seen with protease inhibitors), and, in the future, tumor heterogeneity should be addressed in preclinical stages of drug development.

EXPERIMENTAL PROCEDURES

Cell Culture

Cells were maintained at 37°C/5% CO_2 in Dulbecco's modified Eagle's medium (Sigma) supplemented with 10% fetal bovine serum (Sigma) and 0.5% penicillin-streptomycin (Sigma). Prior to injection, UACC62 cells were stained with CellTrace CFSE Cell Proliferation Kit (Invitrogen). WM266-4 shFN cells were generated with the Block-iT Pol II miR RNAi expression vector kit (Invitrogen). MT1-MMP knockdown in WM266-4 cells was achieved by transfecting 2nM siRNA against MT1-MMP (Dharmacom) using INTERFERin (Polyplus Transfection) transfection reagent.

Xenograft Assay

All animal studies described within were approved by The University of Manchester Ethical Review Board and performed according to UK Home Office regulations. Suspended cells were injected into the pericardial cavity of 48 hr postfertilization zebrafish embryos. Engrafted embryos were maintained at 34°C for 4 days. As needed, embryos were treated at 1 day postinjection (dpi) with DMSO (Sigma) or a cocktail of protease inhibitors or

GM6001 alone (for 72 hr). At 4 dpi, embryos were fixed in 4% paraformaldehyde (PFA, Sigma) in PBS at 4°C overnight.

Whole-Mount Immunofluorescence

Embryos were incubated with anti-collagen I (1:800 dilution, rabbit polyclonal, 1:4,000 dilution; Rockland Immunochemicals) and anti-fibronectin (1:1,000 dilution, mouse monoclonal Ab6328; Abcam) antibodies and then with secondary Alexa Fluor antibodies: anti-mouse 594, anti-mouse 633, anti-rabbit 594, and anti-rabbit 633 (1:150 dilution, Invitrogen).

Microscopy and Analysis

Tumors were imaged at 1 and 4 dpi using a Leica TCS SP5 AOBS upright confocal (Leica Microsystems). Z stacks were processed using Volocity software (Perkin Elmer). All experiments were performed a minimum of three times. Relative invasion is defined as the average number of cells located outside the pericardial cavity at 4 dpi normalized to the average number for the control group. All statistical analysis was performed using GraphPad Prism version 5 (GraphPad Software).

SUPPLEMENTAL INFORMATION

Supplemental Information includes Supplemental Experimental Procedures and five figures and can be found with this article online at http://dx.doi.org/10.1016/j.celrep.2014.06.045.

AUTHOR CONTRIBUTIONS

A.C. helped design and performed the majority of experiments (with support from L.F.D.A. and J.F.) and analyzed data; J.K. performed immunostaining for MITF on patient samples and acquired images; and C.W. and A.H. conceived the study, designed experiments, and analyzed data. All authors contributed to interpreting results and writing the paper.

ACKNOWLEDGMENTS

This work was funded by grants to A.H. and C.W. from the Harry J Lloyd Charitable Trust and Cancer Research UK (ref C11876/A12724 and C11591/A16416) and also by a Wellcome Trust Institutional Strategic Support Fund (ISSF) award (097820/Z/11/A) to The University of Manchester. We thank Dr Imanol Arozarena (The University of Manchester) for generating cell lines expressing fluorescent proteins and providing preliminary data for UACC62 cells, Professor Karl Kadler (The University of Manchester) for many helpful suggestions, and Dr Angeliki Malliri (Cancer Research UK Manchester Institute) for critically reading the manuscript.

Received: November 11, 2013
Revised: May 8, 2014
Accepted: June 23, 2014
Published: July 24, 2014

REFERENCES

Arozarena, I., Bischof, H., Gilby, D., Belloni, B., Dummer, R., and Wellbrock, C. (2011). In melanoma, beta-catenin is a suppressor of invasion. Oncogene *30*, 4531–4543.

Carreira, S., Goodall, J., Denat, L., Rodriguez, M., Nuciforo, P., Hoek, K.S., Testori, A., Larue, L., and Goding, C.R. (2006). Mitf regulation of Dia1 controls melanoma proliferation and invasiveness. Genes Dev. *20*, 3426–3439.

Cleary, A.S., Leonard, T.L., Gestl, S.A., and Gunther, E.J. (2014). Tumour cell heterogeneity maintained by cooperating subclones in Wnt-driven mammary cancers. Nature *508*, 113–117.

Friedland, J.C., Lee, M.H., and Boettiger, D. (2009). Mechanically activated integrin switch controls alpha5beta1 function. Science *323*, 642–644.

Gaggioli, C., Deckert, M., Robert, G., Abbe, P., Batoz, M., Ehrengruber, M.U., Ortonne, J.P., Ballotti, R., and Tartare-Deckert, S. (2005). HGF induces fibronectin matrix synthesis in melanoma cells through MAP kinase-dependent signaling pathway and induction of Egr-1. Oncogene *24*, 1423–1433.

Gaggioli, C., Hooper, S., Hidalgo-Carcedo, C., Grosse, R., Marshall, J.F., Harrington, K., and Sahai, E. (2007). Fibroblast-led collective invasion of carcinoma cells with differing roles for RhoGTPases in leading and following cells. Nat. Cell Biol. *9*, 1392–1400.

Greaves, M., and Maley, C.C. (2012). Clonal evolution in cancer. Nature *481*, 306–313.

Hoek, K.S., and Goding, C.R. (2010). Cancer stem cells versus phenotype-switching in melanoma. Pigment Cell Melanoma Res *23*, 746–759.

Hoek, K.S., Schlegel, N.C., Brafford, P., Sucker, A., Ugurel, S., Kumar, R., Weber, B.L., Nathanson, K.L., Phillips, D.J., Herlyn, M., et al. (2006). Metastatic potential of melanomas defined by specific gene expression profiles with no BRAF signature. Pigment Cell Res. *19*, 290–302.

Hoek, K.S., Eichhoff, O.M., Schlegel, N.C., Döbbeling, U., Kobert, N., Schaerer, L., Hemmi, S., and Dummer, R. (2008). In vivo switching of human melanoma cells between proliferative and invasive states. Cancer Res. *68*, 650–656.

Hou, J.M., Krebs, M.G., Lancashire, L., Sloane, R., Backen, A., Swain, R.K., Priest, L.J., Greystoke, A., Zhou, C., Morris, K., et al. (2012). Clinical significance and molecular characteristics of circulating tumor cells and circulating tumor microemboli in patients with small-cell lung cancer. J. Clin. Oncol. *30*, 525–532.

Kamoshida, G., Matsuda, A., Miura, R., Takashima, Y., Katsura, A., and Tsuji, T. (2013). Potentiation of tumor cell invasion by co-culture with monocytes accompanying enhanced production of matrix metalloproteinase and fibronectin. Clin. Exp. Metastasis *30*, 289–297.

Khoja, L., Shenjere, P., Hodgson, C., Hodgetts, J., Clack, G., Hughes, A., Lorigan, P., and Dive, C. (2014). Prevalence and heterogeneity of circulating tumour cells in metastatic cutaneous melanoma. Melanoma Res. *24*, 40–46.

King, R., Weilbaecher, K.N., McGill, G., Cooley, E., Mihm, M., and Fisher, D.E. (1999). Microphthalmia transcription factor. A sensitive and specific melanocyte marker for MelanomaDiagnosis. Am. J. Pathol. *155*, 731–738.

Levental, K.R., Yu, H., Kass, L., Lakins, J.N., Egeblad, M., Erler, J.T., Fong, S.F., Csiszar, K., Giaccia, A., Weninger, W., et al. (2009). Matrix crosslinking forces tumor progression by enhancing integrin signaling. Cell *139*, 891–906.

Ohanna, M., Giuliano, S., Bonet, C., Imbert, V., Hofman, V., Zangari, J., Bille, K., Robert, C., Bressac-de Paillerets, B., Hofman, P., et al. (2011). Senescent cells develop a PARP-1 and nuclear factor-kappaB-associated secretome (PNAS). Genes Dev. *25*, 1245–1261.

Provenzano, P.P., Eliceiri, K.W., Campbell, J.M., Inman, D.R., White, J.G., and Keely, P.J. (2006). Collagen reorganization at the tumor-stromal interface facilitates local invasion. BMC Med. *4*, 38.

Ramaswamy, S., Ross, K.N., Lander, E.S., and Golub, T.R. (2003). A molecular signature of metastasis in primary solid tumors. Nat. Genet. *33*, 49–54.

Rozenberg, G.I., Monahan, K.B., Torrice, C., Bear, J.E., and Sharpless, N.E. (2010). Metastasis in an orthotopic murine model of melanoma is independent of RAS/RAF mutation. Melanoma Res. *20*, 361–371.

Sabeh, F., Ota, I., Holmbeck, K., Birkedal-Hansen, H., Soloway, P., Balbin, M., Lopez-Otin, C., Shapiro, S., Inada, M., Krane, S., et al. (2004). Tumor cell traffic through the extracellular matrix is controlled by the membrane-anchored collagenase MT1-MMP. J. Cell Biol. *167*, 769–781.

Sahai, E., and Marshall, C.J. (2003). Differing modes of tumour cell invasion have distinct requirements for Rho/ROCK signalling and extracellular proteolysis. Nat. Cell Biol. *5*, 711–719.

Scheel, C., and Weinberg, R.A. (2012). Cancer stem cells and epithelial-mesenchymal transition: concepts and molecular links. Semin. Cancer Biol. *22*, 396–403.

Sottile, J., and Hocking, D.C. (2002). Fibronectin polymerization regulates the composition and stability of extracellular matrix fibrils and cell-matrix adhesions. Mol. Biol. Cell *13*, 3546–3559.

Tsuji, T., Ibaragi, S., Shima, K., Hu, M.G., Katsurano, M., Sasaki, A., and Hu, G.F. (2008). Epithelial-mesenchymal transition induced by growth suppressor

p12CDK2-AP1 promotes tumor cell local invasion but suppresses distant colony growth. Cancer Res. *68*, 10377–10386.

Uhlen, M., Oksvold, P., Fagerberg, L., Lundberg, E., Jonasson, K., Forsberg, M., Zwahlen, M., Kampf, C., Wester, K., Hober, S., et al. (2010). Towards a knowledge-based Human Protein Atlas. Nat. Biotechnol. *28*, 1248–1250.

Vakiani, E., Janakiraman, M., Shen, R., Sinha, R., Zeng, Z., Shia, J., Cercek, A., Kemeny, N., D'Angelica, M., Viale, A., et al. (2012). Comparative genomic analysis of primary versus metastatic colorectal carcinomas. J. Clin. Oncol. *30*, 2956–2962.

Vignot, S., Frampton, G.M., Soria, J.C., Yelensky, R., Commo, F., Brambilla, C., Palmer, G., Moro-Sibilot, D., Ross, J.S., Cronin, M.T., et al. (2013). Next-generation sequencing reveals high concordance of recurrent somatic alterations between primary tumor and metastases from patients with non-small-cell lung cancer. J. Clin. Oncol. *31*, 2167–2172.

Walter, M.J., Shen, D., Ding, L., Shao, J., Koboldt, D.C., Chen, K., Larson, D.E., McLellan, M.D., Dooling, D., Abbott, R., et al. (2012). Clonal architecture of secondary acute myeloid leukemia. N. Engl. J. Med. *366*, 1090–1098.

Wellbrock, C., and Hurlstone, A. (2010). BRAF as therapeutic target in melanoma. Biochem. Pharmacol. *80*, 561–567.

Wolf, K., Mazo, I., Leung, H., Engelke, K., von Andrian, U.H., Deryugina, E.I., Strongin, A.Y., Bröcker, E.B., and Friedl, P. (2003). Compensation mechanism in tumor cell migration: mesenchymal-amoeboid transition after blocking of pericellular proteolysis. J. Cell Biol. *160*, 267–277.

Wolf, K., Wu, Y.I., Liu, Y., Geiger, J., Tam, E., Overall, C., Stack, M.S., and Friedl, P. (2007). Multi-step pericellular proteolysis controls the transition from individual to collective cancer cell invasion. Nat. Cell Biol. *9*, 893–904.

Wolf, K., Te Lindert, M., Krause, M., Alexander, S., Te Riet, J., Willis, A.L., Hoffman, R.M., Figdor, C.G., Weiss, S.J., and Friedl, P. (2013). Physical limits of cell migration: control by ECM space and nuclear deformation and tuning by proteolysis and traction force. J. Cell Biol. *201*, 1069–1084.

Wu, M., Pastor-Pareja, J.C., and Xu, T. (2010). Interaction between Ras(V12) and scribbled clones induces tumour growth and invasion. Nature *463*, 545–548.

Yu, M., Bardia, A., Wittner, B.S., Stott, S.L., Smas, M.E., Ting, D.T., Isakoff, S.J., Ciciliano, J.C., Wells, M.N., Shah, A.M., et al. (2013). Circulating breast tumor cells exhibit dynamic changes in epithelial and mesenchymal composition. Science *339*, 580–584.

SUPPLEMENTAL INFORMATION

Heterogeneous tumour-subpopulations co-operate to drive invasion

Anna Chapman, Laura Fernandez del Ama, Jennifer Ferguson, Jivko Kamarashev, Claudia Wellbrock, and Adam Hurlstone

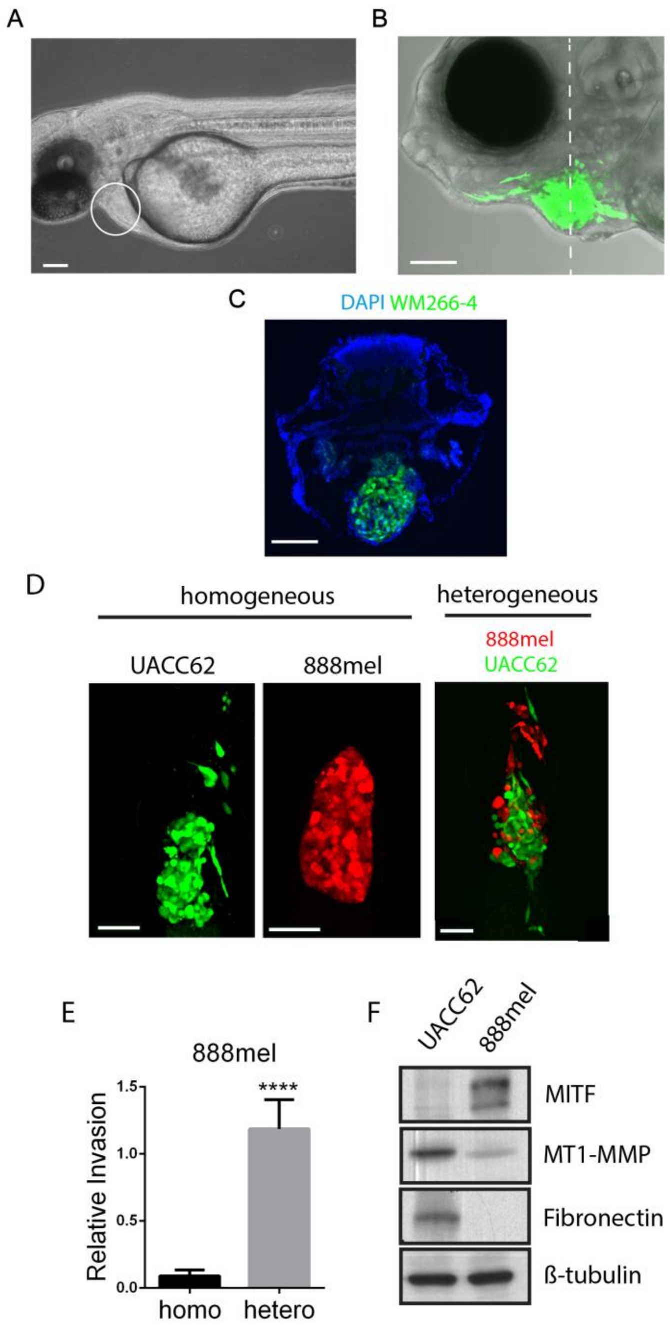

Figure S1. Zebrafish embryo melanoma xenografts. (Related to Figure 1.)

(A) A 48 hour post fertilisation zebrafish showing the pericardial cavity (white circle) as the site of xenograft injection. (B) GFP labelled WM266-4 melanoma cells injected into the pericardial cavity form a tumour-like mass capable of local invasion. Dashed line indicates plane of section in (C). (C) A cryo-section through the tumour like mass shown in (B) was labelled with DAPI (nuclei) and melanoma cells visualised through GFP fluorescence (weak autofluorescence is apparent at certain sites). The mass engrafted onto the body wall of the pericardial cavity. (D) UACC62 and 888mel homogeneous and heterogeneous xenografts. (E) Quantification of 888mel invasion depicted in (D) normalised to UACC62 invasion; mean ± SEM; Mann-Whitney test; **** = $p < 0.0001$; $N \geq 26$ from 3 independent experiments. (F) Western blot showing protein levels of MITF, MT1-MMP and fibronectin in UACC62 and 888mel cells. Scale bars = 100 μm.

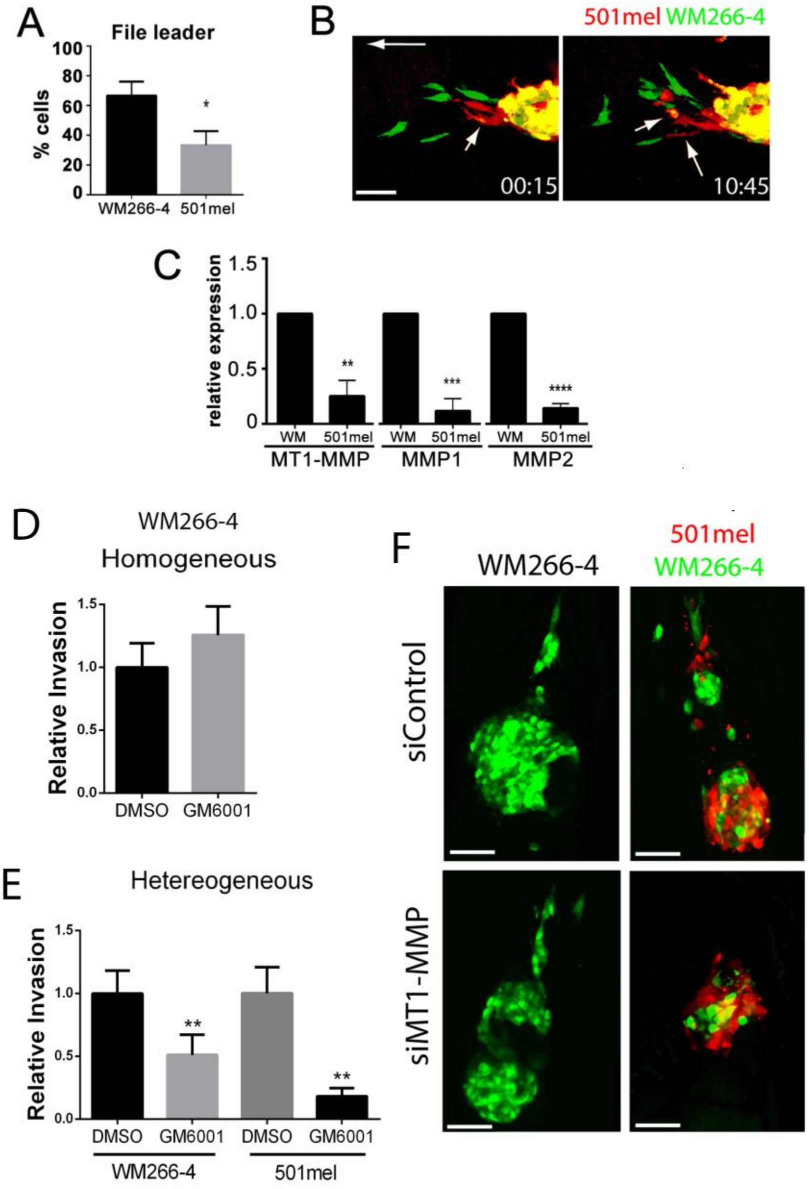

Figure S2. Follower-leader behaviour underlies co-operative invasion in heterogeneous xenografts— a crucial role for MT1-MMP. (Related to Figure 2.)

(A) Quantification of file leader; mean ± SEM; Mann-Whitney test; * = p<0.05; N ≥ 26 from 3 independent experiments. (B) Video time-lapse stills showing WM266-4 'leader' cells and 501mel 'follower' cells (white arrows); scale bar = 50 μm. (C) Relative MMP expression determined by RT-qPCR from 3 independent experiments. Mean ± SEM; unpaired Student's T-test; ** = p <0.01; *** = p < 0.001; **** = p < 0.0001. (D) Quantification of invasion of WM266-4 cells in homogeneous treated with either the vehicle control DMSO or GM6001; mean ± SEM; Mann-Whitney test; N ≥ 14 from 3 independent experiments. (E) Quantification of invasion of WM266-4 and 501mel cells in heterogeneous xenografts treated with either the vehicle control DMSO or GM6001; mean ± SEM; Kruskal-Wallis test followed by Dunn's multiple comparisons test; ** = p<0.01; N ≥ 17 from 3 independent experiments. (F) Representative images of WM266-4 cells transfected with either control or MT1-MMP specific siRNA in homogeneous and heterogeneous xenografts.

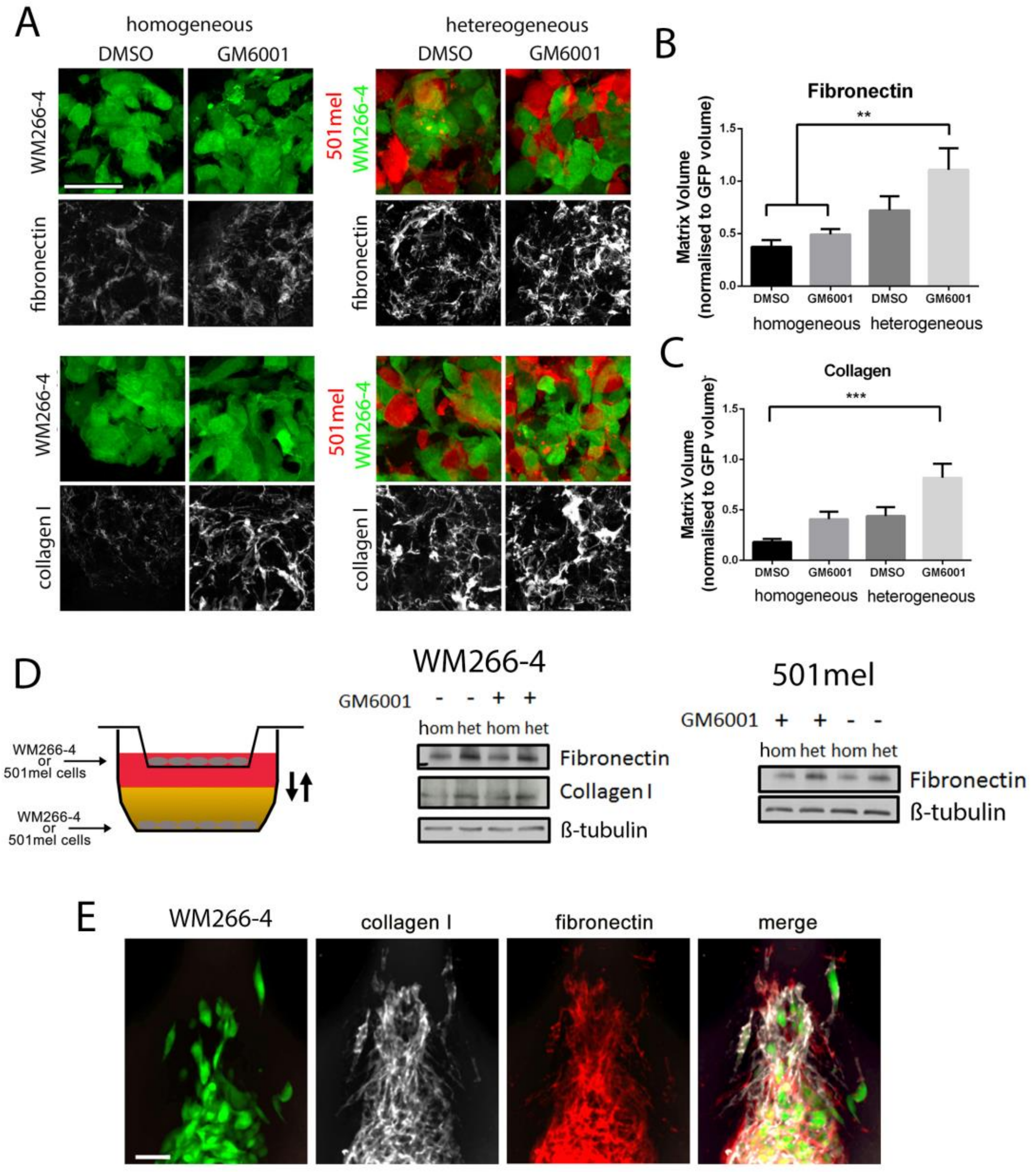

Figure S3. Characterisation of matrix deposition in xenografts. (Related to Figure 4.)

(A) Cryosections of homogeneous or heterogenous xenografts treated either with the vehicle control DMSO, or the pan-MMP inhibitor GM6001 and stained for fibronectin (upper panels) and collagen I (lower panels). (B) Quantification of fibronectin fluorescence intensity volume normalised to WM266-4 (GFP) volume of homogeneous and heterogeneous xenografts; mean ± SEM; one-way ANOVA followed by Tukey's multiple comparisons test; N ≥ 9 from 3 independent experiments. (C) Quantification of collagen I fluorescence intensity volume normalised to WM266-4 (GFP) volume of homogeneous and heterogeneous xenografts; mean ± SEM; Kruskal-Wallis followed by Dunn's multiple comparisons test N ≥ 9 from 3 independent experiments. (D) Western blot showing collagen I or fibronectin expression in either WM266-4 cells (middle panels) or 501mel (right panels) co-cultured either with autologous cells (hom) or heterologous cells (het; as depicted in the cartoon, left). Furthermore, cells were either treated with DMSO or a cocktail of protease inhibitors. (E) Wholemount immunofluorescence labelling of both fibronectin and collagen I in homogeneous WM266-4 xenografts at 4 dpi. Scale bars = 50 μm.

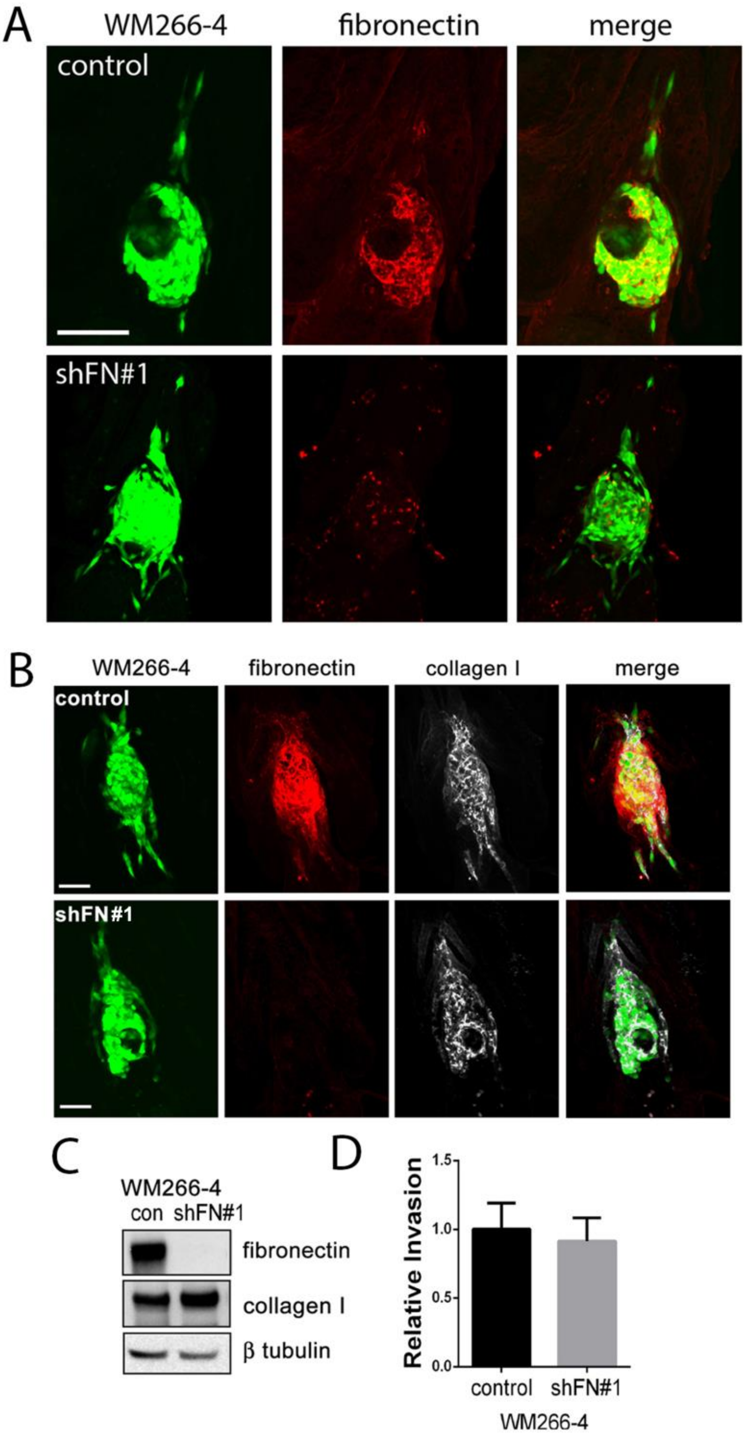

Figure S4. Fibronectin is not required for homogeneous WM226-4 cell invasion and collagen I deposition is independent of fibronectin. (Related to Figure 5.)

(A) Wholemount immunofluorescence labelling of fibronectin in homogeneous xenografts of control (upper panel) and shFN#1 WM266-4 cells (lower panel). Mean ± SEM; unpaired Student's T-test; N ≥ 15 from 3 independent experiments. (B) Wholemount immunofluorescence labelling of fibronectin and collagen I in homogeneous xenografts of control (top panel) and shFN#1 (bottom panel) WM266-4 cells. Scale bars = 100 µm. (C) Western blot showing fibronectin and collagen I expression in control and shFN#1 WM266-4 cells. (D) Relative invasion quantification of (A).

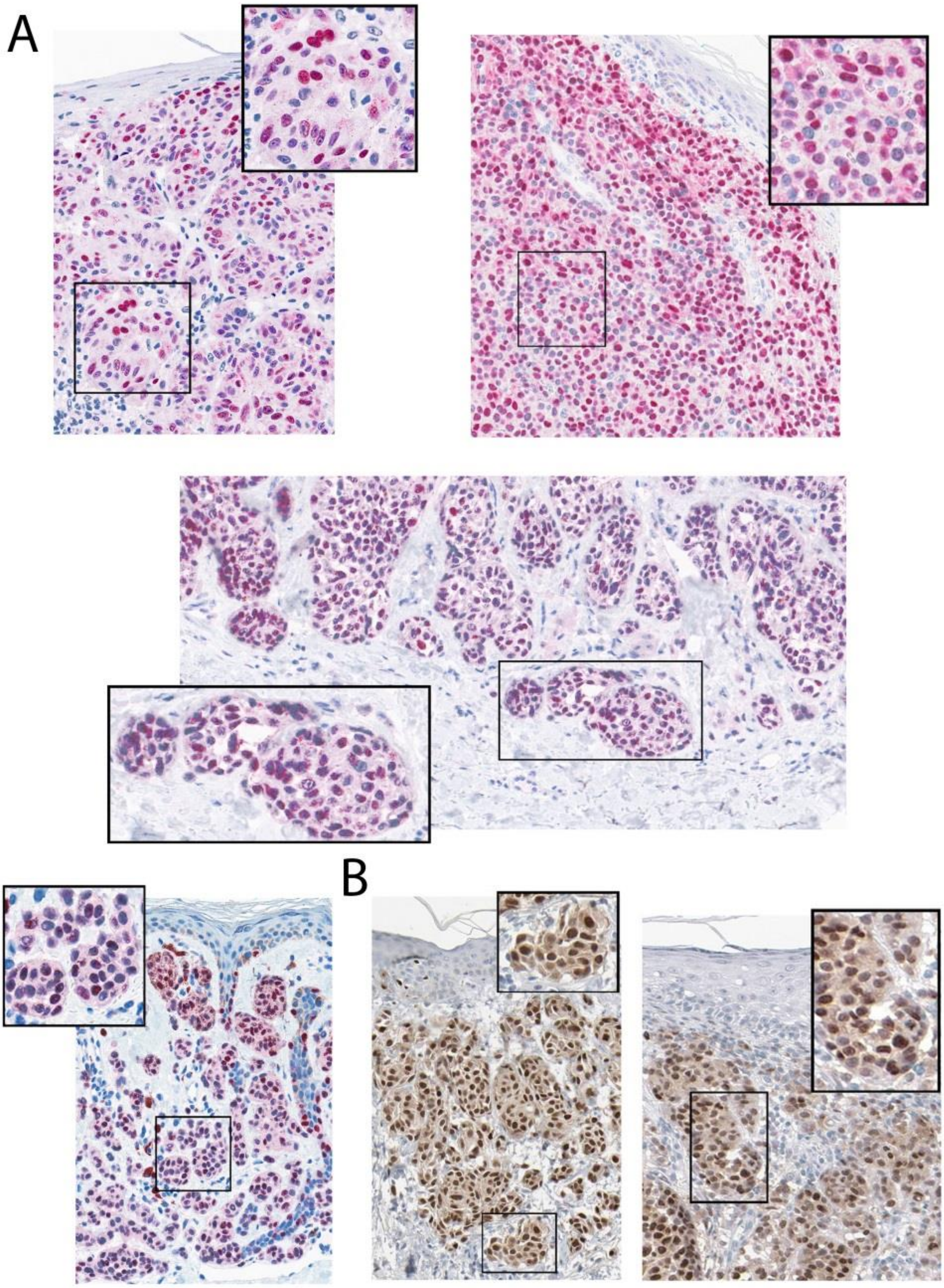

Figure S5. Invading cells are heterogeneous. (Related to Figure 5.)

MITF expression in cells during human melanoma invasion. Boxed area is magnified and reveals heterogeneous MITF expression. (B) Images courtesy of the Human Protein Atlas http://www.proteinatlas.org/ENSG00000187098/cancer/melanoma (Uhlen et al., 2010).

EXTENDED EXPERIMENTAL PROCEDURES

Cell culture

The human metastatic melanoma cell lines UACC62, WM266-4 that express GFP, 501mel and 888 mel that express mcherry were maintained at 37 ^{o}C/5 % CO_2 in DMEM (Sigma) with 10 % fetal bovine serum (Sigma) and 0.5 % penicillin-streptomycin (Sigma). 501mel and 888mel-mCherry cells were maintained in 1mg/ml G418 for selection purposes. Prior to injection, UACC62 cells were stained with 45 µM CellTrace™ CFSE Cell Proliferation Kit (Invitrogen) for 25 minutes in PBS, and washed for 30 minutes in DMEM. Knockdown of MT1-MMP in WM266-4 cells was achieved using INTERFERin (Polyplus Transfection) transfection reagent as per manufacturer's instructions and 2nM siRNA. The MT1-MMP targeted siRNA and scrambled (non-targeting) control siRNA sequences were as follows: MT1-MMP = GAUCAAGGCCAAUGUUCGA; control siRNA AAUAUAAUCACUAUCAGGUGC. Stable fibronectin knockdown (shFN) WM266-4 cells were generated with the Block-iT™ Pol II miR RNAi expression vector kit (Invitrogen) using the pcDNA™ 6.2-GW/EmGFP-miR vector as per manufacturer's instructions. shRNA sequences against fibronectin were as follows:
shFN#1 =
5′-TGCTGACTTCATGTTGTCTCTTCTGCGTTTTGGCCACTGACTGACGCAGAAGACAA
CATGAAGT-3′
and shFN#2 =
5′-TGCTGTGTACTTGGAAATGTGAGATGGTTTTGGCCACTGACTGACCATCTCACTTC
CAAGTACA-3′.

The negative control provided with the kit consists of an insert that forms a hairpin structure and is processed into mature miRNA, but is predicted not to target any known vertebrate gene. WM266-4 cells were transfected with Attractene (Qiagen), cells were then selected using Blasticidin (12 µg/ml; Invitrogen), sorted for GFP expression by FACs Aria (BD Biosciences) and clones produced by limiting dilution.

Melanoma three-dimensional spheroid assay and co-culture system

5000 WM266-4-GFP cells were resuspended in DMEM containing 5% FBS and 1.5% methylcellulose (Sigma) and transferred into a 96 U-well plate for 24 hours to allow spheroid formation. Spheres were then transferred into 2 ml fibrillar bovine dermal collagen (3.1mg/ml stock, 1.9mg/ml final concentration Nucaton) containing 25 µg/ml Fibronectin from Human

Plasma (Sigma Aldrich F0895) in a 6-well plate. 501mel-mCherry or WM266-4 GFP cells plated in 6 well transwell polycarbonate membrane 24mm 0.4um inserts (Appleton Woods) were placed on top of the spheroids embedded in collagen or otherwise on top of monolayers of either 501mel-mCherry or WM266-4 GFP. Either the vehicle control DMSO or a cocktail of protease inhibitors Calpeptin (10 μM, Calbiochem), GM6001 20μM, Enzo Life Technologies), Aprotinin (10μg/ml, Sigma) Leupeptin (10μg/ml, Sigma) were added for 120 hours. Invasion of GFP expressing WM266-4 cells was monitored and images acquired using a Leica DM IL HC inverted microscope and FC340 Cooled Monocrome camera (Leica Microsystems)

RT-PCR

Cell pellets of single 501mel and WM266-4 cells were lysed with Qiazol (Qiagen). RNA was DNase treated (Qiagen) and reverse transcription performed using Omniscript (Qiagen), dNTPs (Qiagen), RNase inhibitor (Biolabs) and random hexamers (Applied Biosystems). RT-PCR was performed using SYBR Green Jumpstart (Sigma) and a Chromo4 qPCR system (BioRad) with triplicate biological repeats for each sample, and fold change calculated normalised to beta-actin expression. Primers were as follows: beta actin Fwd: GCAAGCAGGAGTATGACGAG, Rev: CAAATAAAGCCATGCCAATC, mt1mmp Fwd: AAGCAGCAGCTTCAGCCCCG Rev: GCAGCGATGGCCGCTGAGAG, mmp1 Fwd: CCAGGCCCAGGTATTGGACGGG Rev: TGGGAGAGTCCAAGAGAATGGCCG, mmp2 Fwd: ACCAGCTGGCCTAGTGATGATGT, Rev: GGGGCAGCCATAGAAGGTGTTCA.

Immunoblotting

Cell lysates were prepared using RIPA buffer (150mM NaCl; Sigma, 10mM Tris pH7.2; Sigma, 0.1% SDS, Sigma, 1% Triton-x-100, Sigma, 25mM sodium deoxychoate, Sigma, 5mM EDTA, Sigma) with added protease inhibitors (GE Healthcare). Protein concentration was determined using Pierce ® BCA protein assay kit (Thermo Scientific). Equal lysates were loaded onto a NuPage® Novex® 4-12% Bis-Tris gel (Life Technologies), proteins separated by SDS-PAGE electrophoresis and transferred to nitrocellulose membrane (ImmobilonP) according to standard protocols. Protein bands were detected using primary antibodies against: collagen I (rabbit polyclonal, 1:4000 dilution, Rockland Immunochemicals), fibronectin (rabbit polyclonal 1:5000, F3648 Sigma), beta-tubulin (rabbit polyclonal, 1:4000, Santa Cruz Biotechnologies), mitf (mouse monoclonal 1:1000 , Neomarkers) and anti ERK2 (rabbit polyclonal 1:4000m, Santa Cruz Biotechnologies). Primary antibodies were detected and visualised using an anti rabbit or anti-mouse horse radish peroxidise (HRP)-tagged secondary antibody (1:5000, GE Healthcare) and by the addition of chemi-luminescence substrate (ECL, Perkin-Elmer) and autoradiography.

Xenograft assay

Adult zebrafish (*Danio rerio*) were maintained at the University of Manchester Biological Services Unit according to National Home Office regulations under the Animals (Scientific Procedures) Act 1986. *Casper* strain (*roy*$^{-/-}$, *nacre*$^{-/-}$) zebrafish were used throughout the study to generate embryos completely lacking pigment which can otherwise obscure imaging. WM266-4 GFP, 501mel-mCherry cells, a 1:1 mix of these two cell lines, stained UACC62, 888mel-mCherry or a 2:1 mix of these two cell lines were resuspended at $1.6x10^7$cells/ml on ice with 0.5% polyvinylpyrrolidone K60 solution (PVP, Sigma). 48 hours post fertilisation (hpf) embryos were anaesthetised with 0.1mg/ml MS222 (Sigma) and approximately 500 cells were injected into the pericardial cavity, using a micropipette and pump (World Precision Instruments). Engrafted embryos were maintained at 34°C for 4 days. As needed, embryos were treated at 1 day post injection (dpi) with the vehicle control 0.1% dimethyl sulfoxide (DMSO, Sigma) or a cocktail of protease inhibitors: Calpeptin (1.5μM, Calbiochem), GM6001 (3μM, Enzo Life Technologies), Aprotinin (1.5μg/ml, Sigma) Leupeptin (1.5μg/ml, Sigma) or GM6001 alone (20μM, Sigma) for 72 hours. At 4dpi, embryos were fixed in 4% parafomaldehyde (PFA, Sigma) in PBS at 4°C overnight.

Wholemount immunofluorescence

Fixed 4 dpi zebrafish embryos were washed in PBS, acetone cracked in ice-cold acetone for 10 minutes on ice and blocked for 1 hour in PBDT buffer (PBS with 1 % DMSO, 1 % bovine serum albumin (BSA; Sigma), 0.5 % triton X-100 (Sigma)) and 5 % goat serum (Sigma). Zebrafish were incubated overnight with anti-collagen I (1:800 dilution, rabbit polyclonal; Rockland Immunochemicals) and anti-fibronectin (1:1000 dilution, Ab6328; Abcam) at 4 ^{o}C in PBDT buffer. Zebrafish were washed in PBS with 0.5 % Triton-x-100 (PBST) and incubated with secondary Alexa Fluor antibodies: anti-mouse 594, anti-mouse 647, anti-rabbit 594 and anti-rabbit 647 (1:150 dilution, Invitrogen) for 2 hours in PBDT followed by washes with PBST.

Sectioning

Fixed 4 dpi zebrafish embryos were either cryo-preserved in 30 % sucrose (Sigma) in water overnight before equilibration in OCT for 1 hour, or cryo-preserved in 20% sucrose for 3 hours before equilibration in 15% fish gelatin (Sigma) overnight. Embedded zebrafish embryos were frozen and 12 μm sections were cut using a cryostat (Leica model). Sections were warmed, permeabilised in 1% saponin, incubated overnight with anti-MITF antibody (1:40, Leica), anti-collagen antibody (as before) or anti-fibronectin antibody (as before) and incubated with Alexa-

Fluor anti-mouse 647 as before. Sections were mounted in vectashield with DAPI (VectaLabs) for imaging nuclei.

Microscopy

1 dpi engrafted zebrafish embryos were anaesthetised in 0.1mg/ml MS222. Fixed 4 dpi engrafted zebrafish were mounted in 1.5 % low melting agarose (LMP, Flowgen Biosciences). Tumours were imaged using a Leica TCS SP5 AOBS upright confocal (Leica Microsystems) using a 20 x 0.50 Plan Fluotar dipping objective and 1.5 x confocal zoom. When it was not possible to eliminate cross-talk between channels, the images were collected sequentially. Z stacks from the top to the bottom of the tumour were captured and the maximum intensity projections of these 3D stacks are shown in the results. Zebrafish embryos sections were imaged using Leica TCS SP5 AOBS inverted confocal using a 40 x 0.50 Plan Fluotar objective and 2 x confocal zoom. Z stacks were captured and the maximum intensity projections are shown.

MITF Immunohistochemistry

To check the expression of MITF in human melanoma biopsies, formalin fixed, paraffin embedded archive samples were stained by immunohistochemistry after antigen recovery. Briefly after deparaffinisation, antigen retrieval was performed by the pressure cooker method using EDTA buffer, pH 8.0. Staining was performed using an automated system (Autostainer plus Dako). A monoclonal mouse antibody raised against a N-terminal fragment of MITF protein of human origin (Santa Cruz Biotechnology, sc-56433) was used at a dilution of 1:60.

Analysis

Captured z stacks were processed using Volocity software (Perkin Elmer, Cambridge, UK). All experiments consist of a minimum of three independent repeats. Relative Invasion Index (RII) is defined as the average number of cells invaded outside the pericardial cavity at 4dpi normalized to the average number in a control group. All data sets were tested for normality using D'Agostino & Pearson omnibus normality test. All statistics were based on continuous variables. Comparisons of two data sets were performed using the paired Student's T-Test (parametric) or Mann-Whitney test (non-parametric). Comparisons of more than two data sets were performed using one-way ANOVA followed by Tukey's multiple comparison test (parametric) or Kruskal-Wallis test followed by Dunn's multiple comparison test (non-parametric). Appropriate statistical tests are annotated in figure legends. All statistical analysis was performed using GraphPad Prism version 6 (GraphPad Software Inc).
